# Mechanisms of breast cancer dormancy in bone metastasis

**DOI:** 10.1007/s10585-026-10412-2

**Published:** 2026-06-03

**Authors:** Maria L. Price, Jiabao Zhou, Christine L. Le Maitre, Lewis A. Quayle, Penelope D. Ottewell

**Affiliations:** 1 Division of Clinical Medicine, University of Sheffield, Beech Hill Road, Sheffield, S10 2RX UK; 2 School of Computing and Digital Technologies, Sheffield Hallam University, Howard St, Sheffield, S1 1WB UK

**Keywords:** Breast cancer, Bone microenvironment, Cancer dormancy, Dormant tumour cells, Metastatic disease, Tumour dormancy models

## Abstract

Bone metastasis remains a serious threat to breast cancer patients. This condition arises from the outgrowth of previously dormant tumour cells in this site. Dormant tumour cells are almost impossible to detect in human patents, and these cells acquire “stem like” characteristics rendering them resistant to current cancer therapies. Furthermore, the development of therapies to target this population has proved challenging. The bone marrow is a particularly permissive environment for tumour cell dissemination and dormancy, but the mechanisms regulating this process remain to be completely elucidated. Expansion of our understanding of the mechanisms underlying tumour dormancy is critical to the development of targeted therapies and thus, the prevention, or treatment, of metastatic disease. This review aims to explore mechanisms of tumour dormancy in bone, in detail, focusing specifically on breast cancer dormancy. In addition to subsequent discussion of traditional and new, state of the art, methods of studying dormancy to aid further research efforts.

## Introduction

Metastatic disease is a considerable clinical challenge and is a major cause of fatality in breast cancer patients. The process of metastasis is poorly understood, and patients can often relapse months, years, or decades after removal of the primary tumour. Cancer progression to metastases and dormancy requires a series of key stepwise changes, regulated by the microenvironment. Cells must undergo epithelial-to-mesenchymal transition (EMT) to allow for entry into the vasculature. Circulating tumour cells (CTCs) home to bone via specific signalling axes, adhere to stromal cells and undergo mesenchymal-to-epithelial transition (MET) and extravasation. From here, disseminated tumour cells (DTCs) can have different fates: cell death, dormancy, or further proliferation into metastatic colonies [1–4].

Tumour cell dormancy within bone allows DTCs to reside in a quiescent state for prolonged periods, resistant to therapeutics, with latency periods exhibiting varying lengths, spanning from months to decades [5–7]. Bone metastasis secondary to breast cancer occurs in 10–30% of cases [8, 9], yet the presence of DTCs in the bone marrow is detected in > 60% of patients when autopsied, with tumour dissemination in distal organs thought to occur before clinical detection of the primary tumour [10–16]. Interestingly, non-proliferative DTCs can survive in the bone marrow simultaneous to other organs containing proliferative metastases without becoming proliferative themselves, demonstrating the importance of the tumour microenvironment in regulating dormancy [17]. There are many factors and pathways that have been implicated in the induction of breast cancer tumour dormancy in bone, or reactivation of dormant cells, but key mechanisms remain to be identified, with studies struggling to effectively replicate the dormant microenvironment and often relying on pre-clinical models [2, 5, 18]. This review will outline the current understanding of mechanisms of breast cancer dissemination to the bone, the impact of the bone microenvironment on tumour cells dormancy and the current tools being utilised to study the process, bringing together knowledge in a rapidly advancing field of cancer research.

## Tumour cell dissemination

**Fig. 1:**
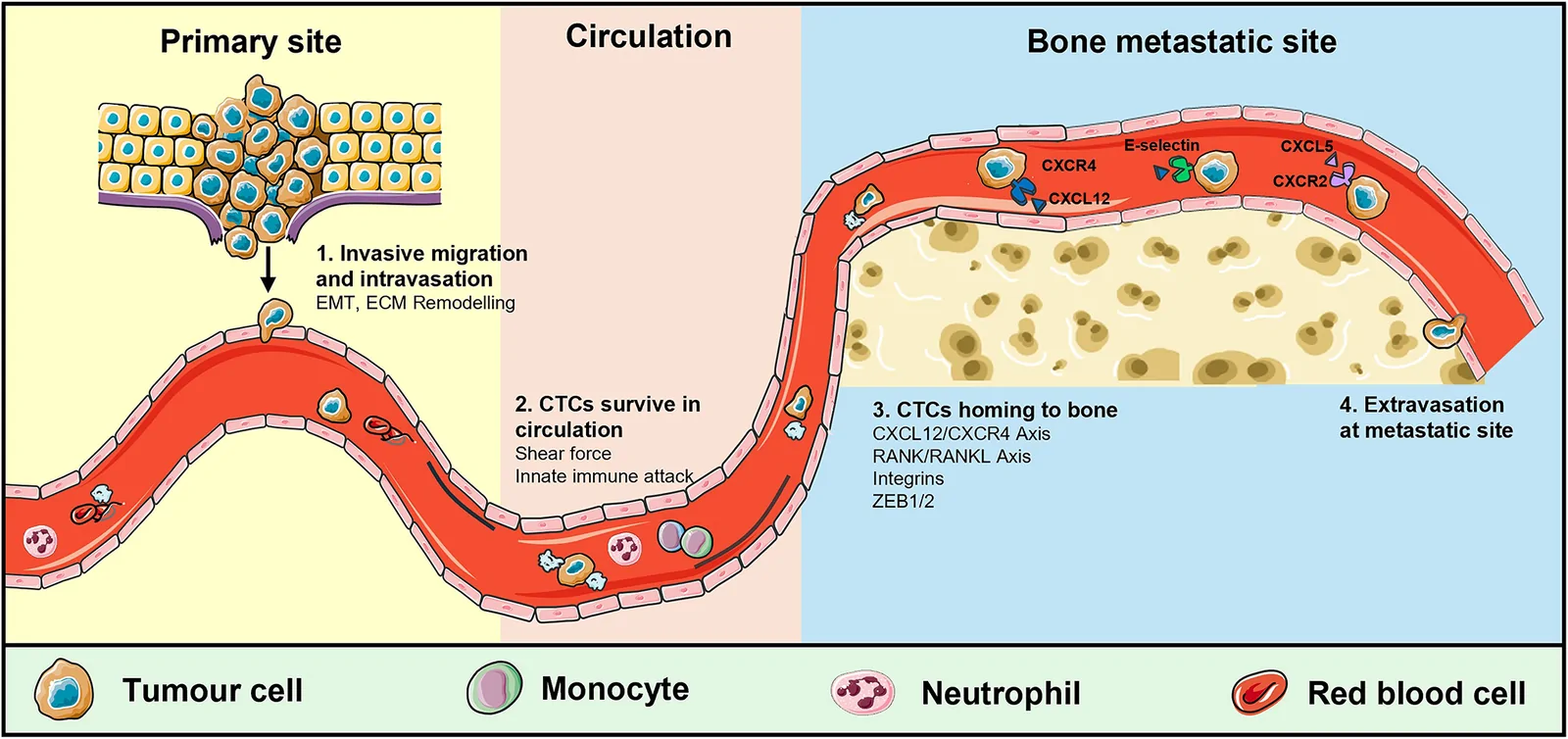
Metastasising cells undergo dynamic changes to adapt to changing environments in what is known as the metastatic cascade [1–4]1. Tumour cells begin the process of metastasis, undergoing epithelial-to-mesenchymal transition (EMT) and remodelling of the extracellular matrix (ECM), before entering blood vessels via intravasation. 2. Circulating tumour cells (CTCs) survive a series of stress factors including matrix detachment, shear forces and immune system attack. 3. CTCs begin homing to the bone, using CXCL12/CXCR4, CXCL5/CXCR2 and E-selectin pathways. 4. CTCs undergo mesenchymal-to-epithelial transition (MET) and extravasation from the blood supply upon reaching the bone, now known as disseminated tumour cells (DTCs). Figure created with adapted images from Servier Medical Art (http://smart.servier.com), licensed under CC BY 4.0 (https://creativecommons.org/licenses/by/4.0/).

Within bone, the different microenvironmental niches act distinctly to impact tumour cell dissemination, dormancy and tumour growth. Many tumours utilise the perivascular niche and haematopoietic stem cell (HSC) homing pathway as footholds to infiltrate the bone marrow. Breast cancer cells have been shown to home to highly osteoblast-populated areas. Anti-resorptive therapy, zoledronic acid (ZOL), alters the endosteal niche and results in breast cancer migration into alternative, osteoblast-rich areas of bone [19], driven by the CXCL12:CXCR4 signalling axis (Fig. 1). Osteoblastic expression of CXCL12 is coupled with CXCR4 expression commonly observed in metastatic breast cancer cells [20–22]. The primary role of CXCL12 in these environments is thought to be establishing the cancer stem cell (CSC) niche, promoting cell survival, proliferation, angiogenesis and metastasis [21, 23, 24]. CXCL12 knock-down in breast cancer cells (BCCs) prevents contact between cells and the bone marrow stroma [25]. E-selectin interactions appear to be key for allowing breast cancer cell entry into the bone marrow, with CXCL12/CXCR4 interactions attaching BCCs to the perivascular niche, using the Wnt pathway, facilitating bone homing and metastasis [23, 26, 27]. In the same chemokine family, CXCL5 is associated with metastatic colonisation, particularly observed in BCCs in mouse models. CXCL5 signalling via its receptor, CXCR2, is sufficient to promote breast cancer cell proliferation and bone colonisation, with inhibition of CXCR2 blocking metastatic cell proliferation [28].

E-cadherin is a fundamental component in regulating metastatic spread, maintaining cell–cell junctions and preventing abnormal cell proliferation. The loss of E-cadherin via genetic or epigenetic silencing is indicative of invasive tumours [29, 30]. Snail family transcriptional repressor (SNAI) 1 and 2, zinc finger E-box binding homeobox (ZEB) 1 and 2, and E47 transcription factors are involved in the repression of E-cadherin, increasing metastatic tumour cell dissemination. In ER + breast cancer, ZEB1/2, vimentin (VIM) and fibronectin 1 (FN1) are upregulated by transcription factors that regulate EMT and maintain a dormant state [31, 32]. When comparing xenografts, triple negative breast cancer (TNBC) cells were shown to proliferate at similar rates in primary and secondary sites, whereas ER + DTCs proliferated slower, exhibited decreased CDH1 (E-cadherin) expression and increased ZEB1/2 expression, taking on a dormant state [31]. In bone specifically, E-cadherin negative breast cancer cells (MDA-MB-231) xenografts resulted in multiple bone metastatic lesions in mice, yet overexpression of E-cadherin resulted in an impaired capacity to form osteolytic metastases [33].

Integrins are cell-surface receptors that bind to components of the extracellular matrix (ECM) and aid in cell proliferation, differentiation, adhesion and migration [34, 35]. Inhibition of integrin β1 results in decreased breast cancer cell migration and adhesion to human bone marrow stromal cells (hBMSCs) *in vitro* [36]. Bone metastasis was similarly reduced in mouse xenograft models exposed to integrin α5 inhibitor, GLPG0187 [37]. Integrin β3 is established to be a key factor in early bone metastasis with involvement in migration capacities in vitro and increased vascular dissemination in vivo. Down-regulation of tumour integrin β3 resulted in impaired bone metastasis of a mammary tumour [38], yet had no effect on lung metastases, highlighting the impact of the metastatic site [39]. Similarly, overexpression of this integrin resulted in increased bone metastasis in vivo following IV injection of the breast cancer cell line, MDA-MB-231, into mice [40]. Of note however, is that tumour integrin β3, rather than stromal integrin β3, proves to be the main driver of this increased migration and metastasis, suggesting the specific microenvironment may not be relevant to its actions [38].

Clinical evidence has shown a positive correlation between *RUNX2* expression and the development of breast cancer bone metastasis [41–43]. Further investigations revealed Runx2 acts to promote bone attraction and adhesion of breast cancer cells in an integrin α5-dependent manner, aiding in tumour cell dissemination [43]. Silencing of integrin α5 results in impaired tumour cell adhesion and migration to fibronectin matrices in vitro, reducing osteolytic bone metastases in vivo, whereas overexpression promoted bone metastasis in vivo [44]. Acting as a heterodimer with integrin β1, α5 (α5β1) orchestrates multiple stages of tumour cell dissemination in the bone, binding with fibronectin in the early stages of metastasis to provide essential adhesion sites for tumour cells [35, 45], and subsequently regulating tumour cell migration and invasion in the bone microenvironment [46, 47]. This evidence reflects the varied roles of integrins on tumour cell adhesion and migration to metastatic sites but does suggest mediated effects of a subset of integrins may be tumour-specific, as opposed to microenvironment-specific. Regardless, integrins remain a potential source of biomarkers for predicting or identifying bone metastasis prior to dormant cell awakening.

## Inducing dormancy

**Fig. 2:**
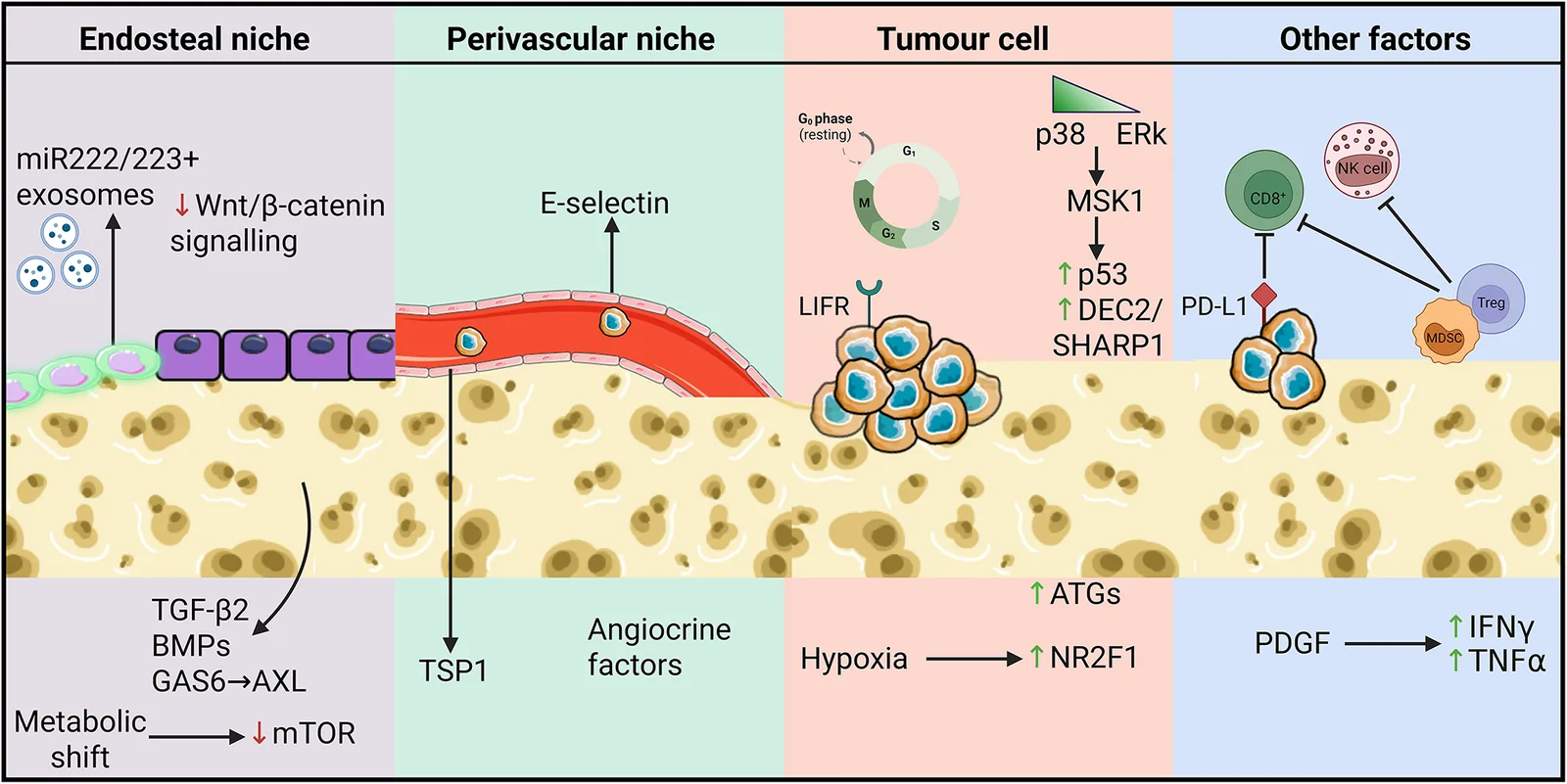
The bone metastatic niches play distinct roles in promoting tumour dormancy, along with processes in the tumour cell itself. DTCs act in the endosteal niche to co-opt the HSC niche, promoting their survival in a dormant state, as well as encouraging MSCs to secrete miR-222/223 + exosomes, maintaining their dormant state. The release of TGFβ2 and BMPs from the bone surface can further promote a quiescent state. Angiogenesis suppressor, TSP1, promotes tumour dormancy from the perivascular niche. Tumour cell intrinsic processes that promote dormancy include a high p38/pErk ratio, increased MSK1 and p53 expression. Immune surveillance is down-regulated by PD-L1 and MDSC/Treg activity [5, 22, 23, 54–57]. Figure created with adapted images from Servier Medical Art (http://smart.servier.com) and BioRender (https://BioRender.com/nyigo7n), licensed under CC BY 4.0 (https://creativecommons.org/licenses/by/4.0/)

MSCs and other cells from the osteoblastic lineage are thought to work within metastatic niches to induce tumour dormancy [48] (Fig. 2). BCCs co-cultured with MSCs exhibited down-regulation of the cancer stem cell marker CD44, resulting in significantly decreased proliferation and increased chemoresistance. Of note, whilst CD44 is used as a marker of cancer stem cells, it is not exclusively expressed by these cells [49]. BCCs in 3D co-culture with MSCs have shown a cancer cell ‘cannibalism’ effect, degrading and internalising MSCs. This resulted in enhanced cancer cell survival whilst also promoting a dormant phenotype through enriched tumour suppressors and pro-survival factors [50]. Tissue spatial heterogeneity has been suggested as one of the drivers for the failure of many anti-tumour therapies. Distinct tumour-niche interactions will occur in small compared to larger tumour lesions, or cells occupying the centre of a tumour compared to the outer, invasive front [45]. This can leave tumour cells at different stages of tumour dormancy, growth and progression. By considering the macroenvironment in addition to the tumour microenvironment, targeting both simultaneously, current therapeutic strategies could be improved [51, 52]. The advancement of spatial transcriptomics methods will significantly improve the knowledge in this field and begin to elucidate the importance of specific cell populations [53].

Recent findings have suggested a key role of exosomes in signalling between breast cancer cells and the metastatic environment. Incubation of exosomes collected from bone marrow-derived MSCs with cancer cells, promoted dormancy-related characteristics including inhibition of proliferation [49]. This is thought to be a result of microRNA (miR-222/223) delivery via exosomes that suppress the expression of a key proliferation-promoting protein [49, 58]. Interestingly, the content of secreted exosomes appears to change following contact with tumour cells, converting to ‘primed’ exosomes and further promoting a dormant phenotype in BCCs [59]. A novel therapeutic strategy targeting dormant BCCs has been proposed, utilising MSCs loaded with antagomiR-222/223, increasing dormant BCC sensitivity to chemotherapy and employing the ‘awakening’ strategy [2, 58]. Furthermore, the study of quiescent BCCs in the bone microenvironment implicated miR-127 and -197 in cell proliferation, transported from the bone marrow to tumour cells via gap junctions or exosomes, acting to reduce CXCL12 levels [60].

Factors secreted by the bone microenvironment, including bone morphogenic proteins (BMPs) can impact cell survival and growth, inducing tumour dormancy. BMPs and growth arrest-specific 6 (GAS6) produced by osteoclasts can directly inhibit DTC 205. Osteoblast-secreted factors, such as BMP7 and transforming growth factor β2 (TGF-β2) bind to DTC receptors and trigger the TGFβRIII– axis. This results in increased p38 activation and subsequent increased p38/ERK ratio, driving dormancy. Increased p38 levels results in increased activation of p53 and DEC2/SHARP1 (also known as BHLHB3) to induce quiescence, further contributing to tumour cell dormancy [61]. TGF-β2 and BMP7 from NG2+/Nestin + MSCs promote HSC quiescence in the BME activating a quiescent pathway via p38, inducing the activation of cell cycle inhibitor, p27 [62]. Mitogen- and stress-activated protein kinase 1 (MSK1), a downstream effector of p38, has been shown to modulate dormancy by modulating the chromatin structure, reducing the expression of key genes required for luminal cell differentiation (GATA3 and FOXA1). In ER + breast cancer patients, low MSK1 is associated with early metastasis and downregulation in vivo impairs breast cancer cell differentiation and increases their bone homing capabilities [63]. The key osteoblast signalling pathway, Wnt/β-catenin, is implicated in the dormancy of cancer cells, yet the mechanisms remain unknown. Wnt5a, a Wnt signalling inhibitor from the osteoblastic niche, is downregulated in invasive breast carcinomas with higher histological grades, suggesting Wnt signalling acts to prevent tumour outgrowth [64]. Wnt5a acts via Seven In Absentia Homolog 2 (SIAH2) to repress Wnt/β-catenin signalling, dependent on the RTK-like orphan receptor (ROR2). Clinically, Wnt5a overexpression is observed in ER + breast cancers with a mutation of *PIK3CA*, seen in approximately 30% ER + breast cancers. All recurrent breast cancer cases in this study exhibited bone metastasis, highlighting the role of Wnt signalling in preventing the outgrowth of DTCs in bone [65].

Oxygen levels in bone typically lie between 2 and 9%, depending on the specific region. Hypoxia (1% O_2_) in primary tumours has been shown to induce a dormant gene programme in DTCs and encourage nuclear receptor subfamily 2 group F member 1 (NR2F1) and hypoxia-inducible factor 1-alpha (HIF1α) to maintain the DTC stem cell state [66–68]. Acting together, NR2F1 and HIF1α maintain elevated expression of cell cycle inhibitor, p27, and down-regulation of cell cycle regulator, CDK4, sustaining DTCs in cell cycle arrest [66, 67]. Hypoxia-induced quiescence is more notable in ER + BCCs compared to TNBC. This is exemplified by ER + breast cancers being predominantly associated with bone metastasis and longer latency periods [69]. Small molecule CDK4/6 inhibitors, such as Palbociclib, are common first-line therapies for metastatic or advanced ER + /HER2− breast cancer, often given in conjunction with an endocrine therapy [70–72]. Whilst the inclusion of CDK4/6 inhibitors can improve the longevity of these therapeutics, emerging evidence suggests prolonged treatment can result in the proliferation of resistant subclones through multiple pathways, as reviewed by Glaviano et al*.* [73]. This is coupled with several off-target effects, including cardiotoxicity and highlights the need for a deeper mechanistic understanding of CDK functions [71, 72, 74].

In addition to low oxygen conditions, tumour cells need to endure limited nutrient supply to enter and remain in a quiescent state, requiring changes to metabolic pathways. Studies show DTCs have a decreased reliance on glycolysis and instead shift to oxidative phosphorylation and fatty acid oxidation pathways. This metabolic shift involves mitochondrial biogenesis, increasing total mitochondrial mass, dependent on AMP-activated protein kinase (AMPK). Peroxisome proliferator-activated receptor-γ coactivator 1α (PGC-1α) acts alongside epigenetic regulation to alter metabolic gene expression and increase mitochondrial DNA [75]. Metabolic interactions between cancer cells and cells in the TME, including CAFs and immune cells, can influence factors such as nutrient availability and the metabolic signalling pathways utilised by dormant cells. Additionally, dormant tumour cells utilise autophagy to ensure proper energy balance, though this is thought to be an intrinsic process, and not microenvironment specific [76–78]. Under normal conditions, autophagy is regulated by the suppression of mammalian target of rapamycin (mTOR). During cellular stress or nutrient deprivation, mTOR kinase activity is suspended, preventing autophagy initiation and facilitating metabolite recycling and energy generation [77, 79].

Immune surveillance and inflammatory factors are key to tumour cell dormancy, both in inducing and promoting escape from dormancy. In tumour mass dormancy, proliferation is balanced by cell death due to immune surveillance from the immune system, utilising CD4^+^ and CD8^+^ T cells, interleukin (IL)-12 and interferon γ (IFNγ) production by NK cells and M1 macrophages [80]. Breast cancer cell secretion of platelet-derived growth factor D (PDGF-D) can be recognised by NK cells and promote the production of IFNγ and tumour necrosis factor (TNF)α, which act to induce dormancy [81]. Breast cancer cells induced into dormancy in this form exhibit enhanced stemness characteristics, suppressed STING signalling and upregulated BACH1/SOX2 expression, which collectively act to defend against NK cell attacks [82]. Dormant tumour cells will also express PD-L1 as part of the immune evasion efforts, allowing them to inhibit T-cell activity and avoid constant surveillance, maintaining their dormant state [83]. Bone marrow naturally has a high baseline of Tregs and myeloid-derived suppressor cells (MDSCs), resulting in reduced NK and T cell activities. Tumour cells exploit this immunosuppressive environment to remain dormant and avoid immune surveillance [54]. Secretion of TGF-β, vascular endothelial growth factor (VEGF) and indoleamine-2,3-dioxygenase (IDO), amongst other factors, by tumour and stromal cells can induce this MDSC-dependent immunosuppression, with IDO thought to be used by TNBC to reduce local levels of tryptophan, leading to production of cytotoxic metabolites [84].

Histone deacetylase (HDAC) inhibitors are implicated in breast cancer dormancy, acting to up-regulate leukaemia inhibitory factor receptor (LIFR) and promote a dormant phenotype [85–87]. Clinical trials investigating the effect of HDAC inhibitor treatment alongside standard of care chemotherapeutics are underway for advanced breast cancer [88], however early results are not promising, with HDAC inhibitors being reported to have no effects on progression free survival for patients with advanced breast cancer [89–91]. Subsequent pre-clinical studies have revealed an osteolytic effect of HDAC inhibitors in breast cancer bone metastasis models [85], suggesting that these compounds could stimulate release of bone bound growth factors, driving the vicious cycle of bone metastasis, thereby, negating any direct pro-dormancy effects on cancer cells in bone [86]. When combined with ZOL, HDAC inhibitor-driven bone loss was reduced, but not fully corrected, leaving the role of HDAC inhibitors unclear in breast cancer dormancy in bone [85, 92]. MALAT1, acting via Tead3, has also been implicated in the suppression of bone metastasis, impairing Nfatc1-drive osteoclast differentiation, inhibiting bone resorption, tumour awakening and metastasis [93]. However, as with many epigenetic changes in breast cancer bone metastasis, limited evidence exists of its role in dormancy [94].

## Escaping dormancy

**Fig. 3:**
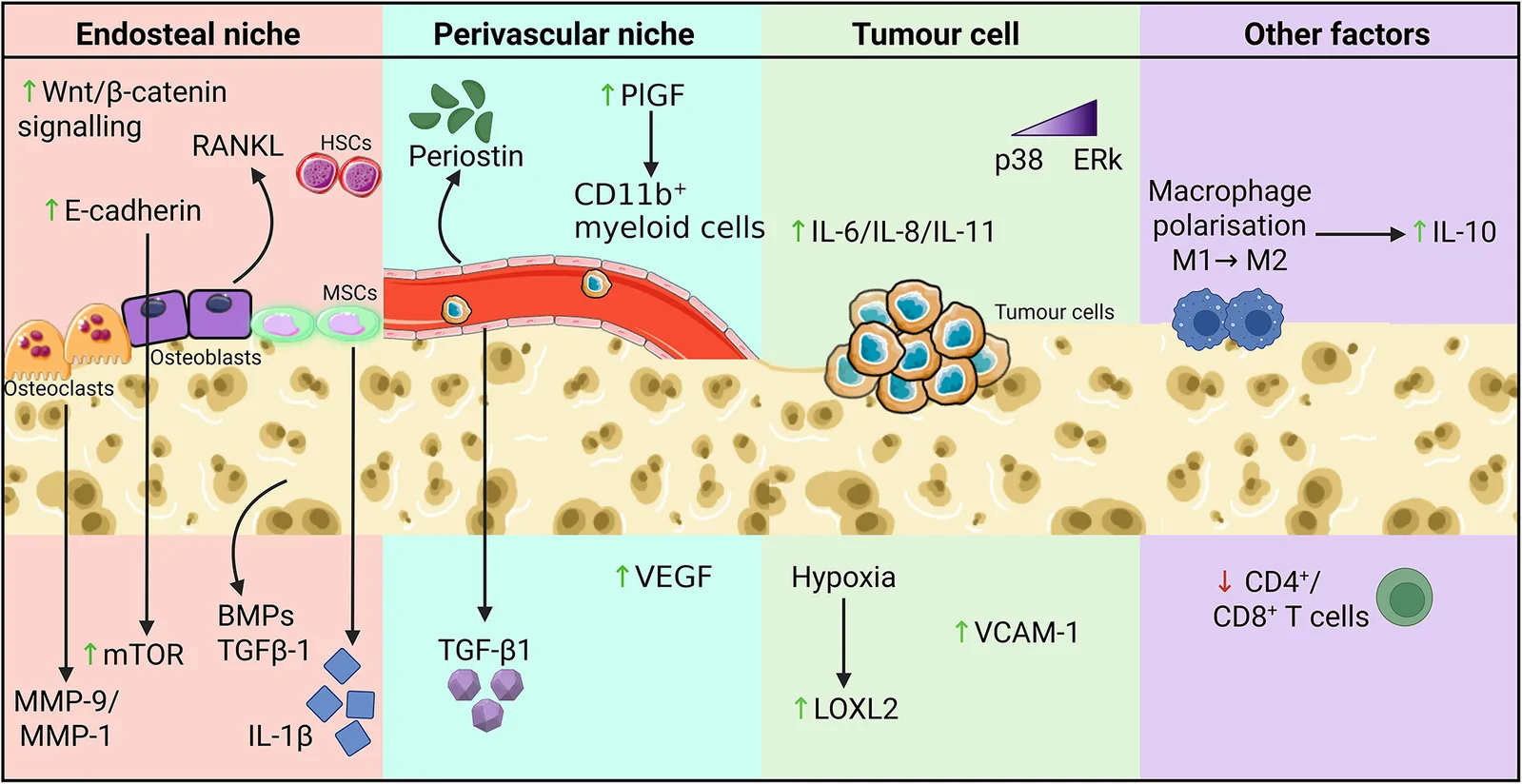
The different bone metastatic niches will enact multiple effects on DTCs to promote an escape from dormancy. In the perivascular niche, angiogenesis promotes metastatic outgrowth by stimulating the release of periostin, with VEGF and PlGF acting alongside. The endosteal niche is responsible for the release of several factors thought to promote tumour micrometastasis, including TGFβ-1, BMPs and IL-1β. Tumour cells interact with osteoblasts to promote E-cadherin expression and increase mTOR activity, promoting tumour outgrowth. In the tumour cells, hypoxia can induce LOXL2 activity and as such, EMT, promoting invasive properties of tumour cells. This is coupled with a lower p38/pErk signalling ratio and increased VCAM-1 activity. Macrophage polarisation from M1 to M2 similarly promotes tumour reawakening via IL-10 [5, 22, 23, 55, 57, 109, 110]. Figure created with adapted images from Servier Medical Art (http://smart.servier.com) and BioRender (https://BioRender.com/ebe4wg0), licensed under CC BY 4.0 (https://creativecommons.org/licenses/by/4.0/)

Tumour cells enter dormancy to enable survival until a time in which the conditions are favourable to proliferate. Once these conditions are met, mechanisms will begin to induce cell escape from dormancy (Fig. 3) [5, 18, 95]. Tumour dormancy is linked to osteoblast activity, whereas osteoclast-mediated mechanisms are thought to induce escape from dormancy and tumour growth. The recruitment of osteoclast precursors and increase in osteoclast activity, driven by vascular cell adhesion protein 1 (VCAM-1) has been shown to promote an escape from dormancy in dormant metastatic breast cancer cell lines [96]. Disruption of the canonical receptor activator of NF-κB/ligand (RANK/RANKL) interactions in osteoclasts with RANKL antagonist, osteoprotegerin-Fc (OPG-Fc), inhibits the growth of dormant DTCs *in vivo* [97]. Bisphosphonates have been widely used to improve symptoms of bone metastasis, reducing bone pain, skeletal fractures and hypercalcemia occurrence. They also have the potential to limit tumour reawakening and expansion, as seen with bisphosphonate treatment reducing in vivo breast cancer tumour growth in bone, despite the presence of DTCs remaining [98–100]. A meta-analysis of cancer risk and bisphosphonates revealed a significantly reduced risk of breast cancer with bisphosphonate treatment [101]. This is further supported by various clinical studies [102–107], including the AZURE trial, whereby adjuvant treatment of breast cancer patients with bisphosphonates inhibits bone metastases and improves disease-free survival in post-menopausal patients [108].

RANKL-inhibitor Denosumab is similarly under investigation for the prevention of tumour cell reawakening in breast cancer bone metastasis and has shown superior responses in delaying skeletal-related events (SREs) compared to ZOL, in advanced breast cancer patients [103, 111–113]. However, a large cohort study in high-risk early breast cancer patients demonstrated no changes in disease-free survival following Denosumab therapy, compared to placebo [114], suggesting the mechanisms used by bisphosphonates to maintain dormancy in bone is not regulated by RANKL and its mechanism remains to be determined [111]. The effects of TGF-β on osteoblast, osteoclasts and bone remodelling are complex and are both spatial and temporal-dependent. While TGF-β2 signalling appears to have a pro-dormancy effect, TGF-β1 can promote bone metastasis through activation of specific genes, utilising the TGFβ-Smad signalling pathway. TGF-β2 signalling via Smad results in the production of several pro-osteolytic factors including IL-11, matrix metalloproteinase 1 (MMP-1), CXCR4 and parathyroid hormone related protein (PTHrP) [115, 116]. An animal model of breast cancer bone metastasis revealed the presence of active TGFβ-Smad signalling, specifically in the bone, with reduced bone metastases upon knockdown of Smad4 [117]. Coco, a secreted antagonist of TGF-β ligands, has recently been suggested as a key regulator of metastatic dormancy and reactivation of BCCs, blocking interaction of BMPs with their receptors. However, these effects are thought to be microenvironment specific, with Coco inducing a gene expression signature strongly associated with metastatic relapse to the lung, but not to the bone or brain [118].

Hypoxia has been discussed earlier in this review as a pro-dormancy microenvironmental trait [67, 68]. However, hypoxia can also be linked to an escape from dormancy. Conditional hypoxia induces lysyl oxidase-like 2 (LOXL2) expression in MCF-7 cells with dormant MCF-7 cells expressing LOXL2 having increased proliferation in the bone and exhibiting increased expression of the oestrogen receptor. Clinically, increased LOXL2 expression is associated with a decrease in relapse-free survival of breast cancer patients. Increased LOXL2 mRNA levels also correlated with increased EMT and stem cell markers, facilitating invasion of surrounding vasculature and hence, metastatic outgrowth [119]. HIF-1α mechanisms that have been determined to promote tumour dormancy can similarly be involved in tumour escape from dormancy and expansion, utilising VEGF production to promote angiogenesis and overcome the oxygen shortage. HIF-1α can also impact immune surveillance to promote tumour growth by suppressing T-cells from killing tumour cells, facilitating further growth [120]. The duration of hypoxia is thought to impact whether dormancy is promoted, or tumour growth. However, the specific timeline that results in this shift is unclear [121, 122]. Mice exposed to acute cyclic hypoxia (12 cycles, 10 min on, 10 min off, 8% O_2_) exhibited increased metastases [123], with intermittent hypoxia (6 cycles, 30 min on, 30 min off, 0%) similarly resulting in promoting an invasive breast cancer phenotype in vitro compared to chronic hypoxia (6 h, 0%) [124]. Of note however, is the varying time frames and conditions used for intermittent hypoxia experiments, ranging from 8 h 3x/week, to 24 cycles of 30 min normoxia/hypoxia, utilising hypoxic oxygen concentrations of 0.1% to 2%, yet all resulting in increased breast cancer progression phenotype [124]. Conversely, prolonged hypoxia (24–48 h, 0.5% O_2_) diminishes LIFR, increasing IL-6 expression and downregulation of dormancy-, quiescence- and cancer stem cell-associated genes [87, 125]. Similarly, cancer cells incubated under chronic hypoxia conditions (16–48 h, 0–0.02% O_2_) showed a more invasive phenotype, yet it is suggested that chronic hypoxia can also render tumour cells incapable of replication following reoxygenation, preventing tumour outgrowth [126]. A key theme throughout studies of hypoxia in tumour dormancy is the variability in conditions used and the labels applied to these e.g. acute vs chronic, limiting the ability to draw a definitive conclusion [124, 127].

MMPs are a family of enzymes that play a key role in the breakdown and remodelling of the ECM and have been implicated in tumour dormancy. MMP-9 is highly expressed by osteoclasts and utilised for osteoclast migration and resorption. High levels of MMP-9 are observed in BCCs, acting in an integrin-αvβ3-dependent manner to promote the migration of MDA-MB-435 cells from bone metastatic sites in mice [128]. MMP13 is also linked to tumour cell awakening, with inhibitor (5-(4-{4-[4-(4-fluorophenyl)-1,3-oxazol-2-yl]phenoxy}phenoxy)-5-(2-methoxyethyl) pyrimidine-2,4,6(1H,3H,5H)-trione (Cmpd-1)) preventing primary tumour growth and reducing the onset of osteolytic lesions following intra-cardiac and intra-mammary injections of MDA-MB-231 cells in mouse models. No effects were observed in soft organ metastases, suggesting MMP13 regulation of dormancy maybe specific the bone microenvironment, and highlighting a potential therapeutic target of interest [129].

Epigenetic regulation of tumour cells plays a role throughout the stages of dormancy. Biopsies from bone metastatic breast cancer patients revealed promotor methylation and reduced expression of High in Normal 1 (*HIN-1*), Retinoic Acid Receptor Beta (*RAR-β*), and Ras Association Domain Family Member 1A (*RASSF1A*) [130], compared to disease-free tissue, all linked to cancer cell plasticity and EMT [130, 131]. This suggests hypermethylation of these tumour suppressors could act as a potential biomarker for bone metastatic progression, improving detection and therapeutic options. Enhancer of zest homolog 2 (EZH2), acting via H3K27me3, also causes changes in gene transcription and epigenetic reprogramming of breast cancer cells in the bone, enhancing their metastatic spread from bone. EZH2 inhibition and knockdown in vivo resulted in impaired metastasis to secondary organs, yet this was after bone lesions were already observed [132]. This highlights the epigenome as a dynamic regulator of tumour cell behaviour but also suggests its primary role may be the regulation of metastasis from the bone, rather than the re-awakening of dormant cells in the bone [94].

Interactions between tumour cells and surrounding bone, stromal and endothelial cells plays an important role in mediating the angiogenic switch. Bone-marrow derived endothelial cells produce high amounts pro-angiogenic factors, including Id-1. Reduced production of these factors significantly reduces the formation of micrometastases in mouse breast cancer models [55, 133]. The release of VEGF from the bone environment promotes tumour angiogenesis, increases oxygen and nutrient delivery, and allows for tumour growth [55, 134, 135]. VEGF action has also been linked to promoting escape from tumour dormancy in diet-induced obesity (DIO) mouse models. VEGF is up-regulated alongside lipocalin-2 (LCN2) and basic fibroblast growth factor (bFGF) with increased tumour frequency and reduced tumour latency observed, bridging the link between obesity and the severity of post-menopausal breast cancer [7]. Placenta growth factor (PlGF) production by BCCs triggers the recruitment of CD11b^+^ myeloid cells from the bone marrow and increases angiogenesis. Breast cancer patients with increased plasma PlGF levels exhibit increased metastasis and a poorer prognosis, implicating PlGF in the outgrowth of breast cancer cells in bone metastasis by overcoming angiogenic dormancy [136].

The bones natural immunosuppressive nature aids in tumour cell evasion of immune clearing. However, when tumour cells are awakening from dormancy, the release of immune suppressive cytokines triggers macrophage polarisation, from M1 to M2, also known as tumour-associated macrophages (TAMs) [109, 110]. M2 macrophages are characterised by increased production of IL-10 and TGF-β, altering the activation of CD4^+^ and CD8^+^ T cells [137, 138]. Other interleukins are also implicated in tumour growth in bone. IL-8 and IL-11 release from breast cancer cells has been shown to increase osteoblastic RANKL production and mediate direct effects on promoting bone metastasis *in vivo* [117, 139]*.* IL-1β has been strongly linked to the reawakening of dormant DTCs via activation of Wnt signalling in the tumour cells, regulating changes to the bone microenvironment [140]. In BCCs, this action of IL-1β has been linked to the demethylating actions of ten-eleven translocation proteins (TETs), whose inhibition results in reduced EMT and bone metastasis markers [141]. Inhibition of IL-1β signalling in mouse models results in decreased osteoblast and osteoclast activity and inhibition of metastatic outgrowth of breast cancer cells in bone, whilst also exhibiting effects on anti-tumour immune cells in an innate response [142, 143]. Conversely, tumour cells expressing high levels of IL-1β increase osteoclast activity, and metastatic outgrowth in mouse bone [144, 145]. From a clinical perspective, higher expression of IL-1β in primary tumours is associated with bone metastasis in breast cancer patients (37% cases with IL-1β vs 5% cases without). Interestingly, this link is independent of ER receptor status in tumours suggesting that IL-1β may be a useful biomarker for predicting future relapse in bone [144, 146]. Additionally, inhibition of IL-1β or it’s receptor IL-1R have been proposed as potential treatment strategies to prevent IL-1β-mediated escape from dormancy [2, 147]. By stimulating bone resorption, these factors allow for the release of pro-tumour growth factors, promoting tumour reawakening.

## Tumour progression

**Fig. 4:**
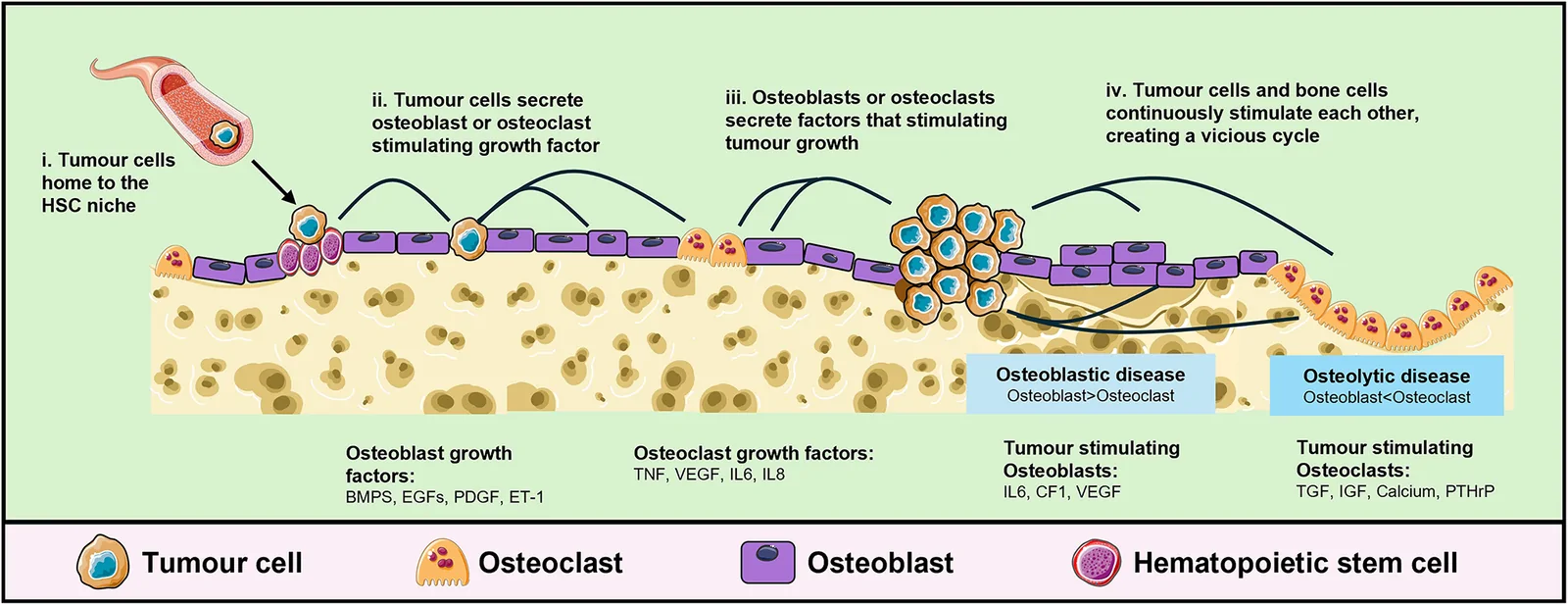
The bone marrow is a common site of tumour metastasis with the microenvironment being a major contributor to tumour growth characteristics. Tumour cells will home to the HSC niche in the bone microenvironment and secrete growth factors to promote osteoblast and osteoclast activity. Increased bone cell activity will result in increased secretion of tumour growth-promoting factors, creating a positive feedback loop, termed the vicious cycle [1, 2, 4, 23]

As discussed, increased osteoclast activity is associated with an escape from dormancy and progression to metastasis. In bone specifically, DTCs release PTHrP, inducing osteoblast-mediated up-regulation of RANKL and down-regulation of OPG [67, 97, 148]. Increased TGF-β release from resorbed bone enables cancer cell proliferation and acts to further increase PTHrP from tumour cells via the activation of SMAD and p38 MAPK pathways, stimulating further osteoclast differentiation, bone resorption and TGFβ release [149]. Clinically, PTHrP expression increases from ~ 60% in primary tumours to 92% in cases of breast cancer bone metastasis, compared to 17% in tumours metastasised to other sites [148, 150–152]. TGFβ also promotes the expression of tumour-growth-promoting cytokine, IL-6, from stromal cells and osteoblasts via Jagged1, further promoting the cancer cell proliferation [23, 153]. This process results in a self-sustaining positive-feedback loop called the ‘vicious cycle’ (Fig. 4) [1, 2, 4, 154]. Further contributions to the ‘vicious cycle’ are observed by integrin α5β1, through modulation of bone turnover. Tumour cells exploit α5β1-signalling to secrete TGFβ and RANKL, disrupting the bone remodelling balance [44, 155]. Circular RNA, circIKBKB, has also been shown to upregulate RANKL and PTHrP, promoting breast cancer metastasis and tumour awakening [156]. Targeting circIKBKB and related signalling components could present a novel therapeutic strategy for halting the vicious cycle and preventing tumour progression in the bone. A key factor with tumour dormancy is the potential for residual disease to re-enter dormancy. There is currently no cure for bone metastasis, and while therapies may reduce the tumour burden on the bone, the risk of further dormant cells reactivating is ever-present, further complicating therapeutic efforts.

Figure created with adapted images from Servier Medical Art (http://smart.servier.com), licensed under CC BY 4.0 (https://creativecommons.org/licenses/by/4.0/).

## Methods to study bone metastasis

The extent of mechanisms involved in tumour dormancy and metastasis in the bone exemplifies the complexity of developing therapeutics. This is further complicated by the limitations of models available to reflect dormancy (Table 1). Many techniques have been developed for cancer research or bone studies but not yet applied to dormancy. In vitro models are commonly used in more preliminary stages for cancer studies to understand cell to cell signalling, impacts on gene expression or signalling pathways. Several 3D tumour cell/bone cell co-culture models of dormancy have been generated over time to improve these early experiments [55, 157–161]. However, these models lack the presence of mineralised bone, ECM components and other cell types, such as endothelial, stromal or immune cells, lacking observation of these interactions. An advancement on cell line 3D cultures is the development of patient-derived organoid (PDO) models, whereby organoids maintain their in vivo genetic and phenotypic heterogeneity, as well as patient-specific drug sensitivities, but these have not yet been incorporated into bone dormancy research [162, 163].

**Table 1:** Experimental methods for the study of bone metastasis and tumour dormancy

	Technique	System Type	Output	Strengths	Limitations
In vitro	2D In Vitro Co-culture Systems [17, 55, 87, 164–166]	Monolayer co-culture of tumour cells with bone marrow cells	Cell-to-cell interactions, dormancy-associated signalling and gene expression (e.g., p38/ERK ratio)	Highly controlled; possibility for genetic manipulation; cost-effective	Lacks bone microenvironment context and 3D architecture
	3D In Vitro Models (Spheroids, Organoids, Scaffold-based Systems) [55, 157–161, 167–170]	Tumour spheroids embedded in scaffolds formed from synthetic polymers, natural fibres or hydrogels, co-cultured with bone marrow cells	ECM stiffness; integrin signalling; hypoxia	3D architecture; reflects mechanical cues; better mimics quiescent phenotype; adjustable stiffness	Limited immune and vascular components; simplified niche
	Bone-on-a-Chip / Microfluidic Platforms [171–177]	Engineered microfluidic bone-mimetic systems	Perivascular niche dynamics	Reproducible; real-time monitoring; adjustable microenvironment; cost-effective (compared to in vivo)	Technical complexity; limited long-term modelling; expensive start up costs
Ex vivo	Ex Vivo Bone Explants [178–181]	Murine or human bone fragments cultured with tumour cells	Native bone matrix with full trabecular structure	Preserves physiological ECM; retains bone-resident cells	Short culture lifespan; limited immune components
In vivo	Syngeneic Mouse Models [87, 135, 182, 183]	Murine tumour cells in mice	Immune-mediated dormancy and escape	Intact immune system; complete niches present	Species-specific tumour biology; fewer bone-specific models
	Xenograft Mouse Models [63, 87, 97, 100, 182]	Human breast cancer cells in mice	Bone invasion and metastatic growth	Human tumour biology; established protocols; longitudinal monitoring ()	May favour overt metastasis over dormancy; species-specific bone biology
	Patient-Derived Xenografts (PDX) [167, 184]	Primary patient tumour implanted into immunodeficient mice	Preserves tumour heterogeneity; clinically relevant dormancy traits	Highly translational; maintains genomic complexity	Expensive; low throughput; immune-deficient host; species-specific bone biology
	Intravital Imaging (IVI) [185–189]	Live imaging in bone windows	Real-time DTC behaviour; cell–niche interactions	Direct visualisation of dormancy; spatial resolution	Surgical expertise required; limited field of view; limited scope to perivascular niche
In silico	Computational modelling [190–192]	Integration of biological findings with computational measurements and calculations	Molecular interactions, cell-niche interactions	Reproducible; complete niche inclusion; adjustable parameters	Inability to process native physiology; complex analysis
*Downstream Analysis*	Single-Cell RNA Sequencing (scRNA-seq) [87, 132, 193]	Transcriptomic profiling of isolated DTCs and niche cells	Cellular heterogeneity; dormancy gene signatures	High-resolution molecular profiling; complete pathway discovery	Lack of spatial context; limited analysis of rare cells; expensive; complex analysis
	Spatial Transcriptomics [42, 53, 193]	Spatial gene expression in bone sections	Localisation of DTCs within niches; tissue spatial heterogeneity	Maintains spatial context; niche mapping	Lower single-cell resolution (platform-dependent); expensive; limited standardised analysis pipelines

Organ-on-a-chip solutions are being advanced in many fields, including bone [69, 171–173]. These systems are advanced in modelling the perivascular niche compared to other in vitro or ex vivo methods. However, they are limited by their timeline, typically lasting for 2–4 weeks, preventing a true understanding of clinical dormancy timescales [171, 172]. Conversely, in vivo models can better reflect these timescales. Breast cancer dormancy in bone has been successfully exhibited in xenograft mice following intra-cardiac injection of human tumour cells [97, 100]. Xenografted mice can often succumb to the results of primary tumours before metastasis and dormancy occurs As such, models have been developed to transplant cells into the orthotopic site and surgically remove tumours once they reach a certain size limit, allowing for subsequent metastasis development and monitoring [194, 195]. Patient-derived xenografts (PDX) improve further compared to the use of tumour cell lines in vivo, better reflecting clinical dormancy traits, but are still subject to species-specific differences [167, 168, 184].

With tissue spatial heterogeneity growing as an area of interest, the impact of spatial transcriptomics is similarly growing [42, 53, 193]. Recent advances have allowed spatial transcriptomics approaches to be applied to the study of human bone [169], but not yet dormancy, with current data sets lacking sufficient sample numbers for conclusive interpretation [53, 169, 193]. As with all experimental setups, it is important to utilise a range of models to draw informed conclusions about DTC behaviour in bone, further helping with the effective delivery of therapeutics.

## Therapeutic approaches for tumour dormancy

**Fig. 5:**
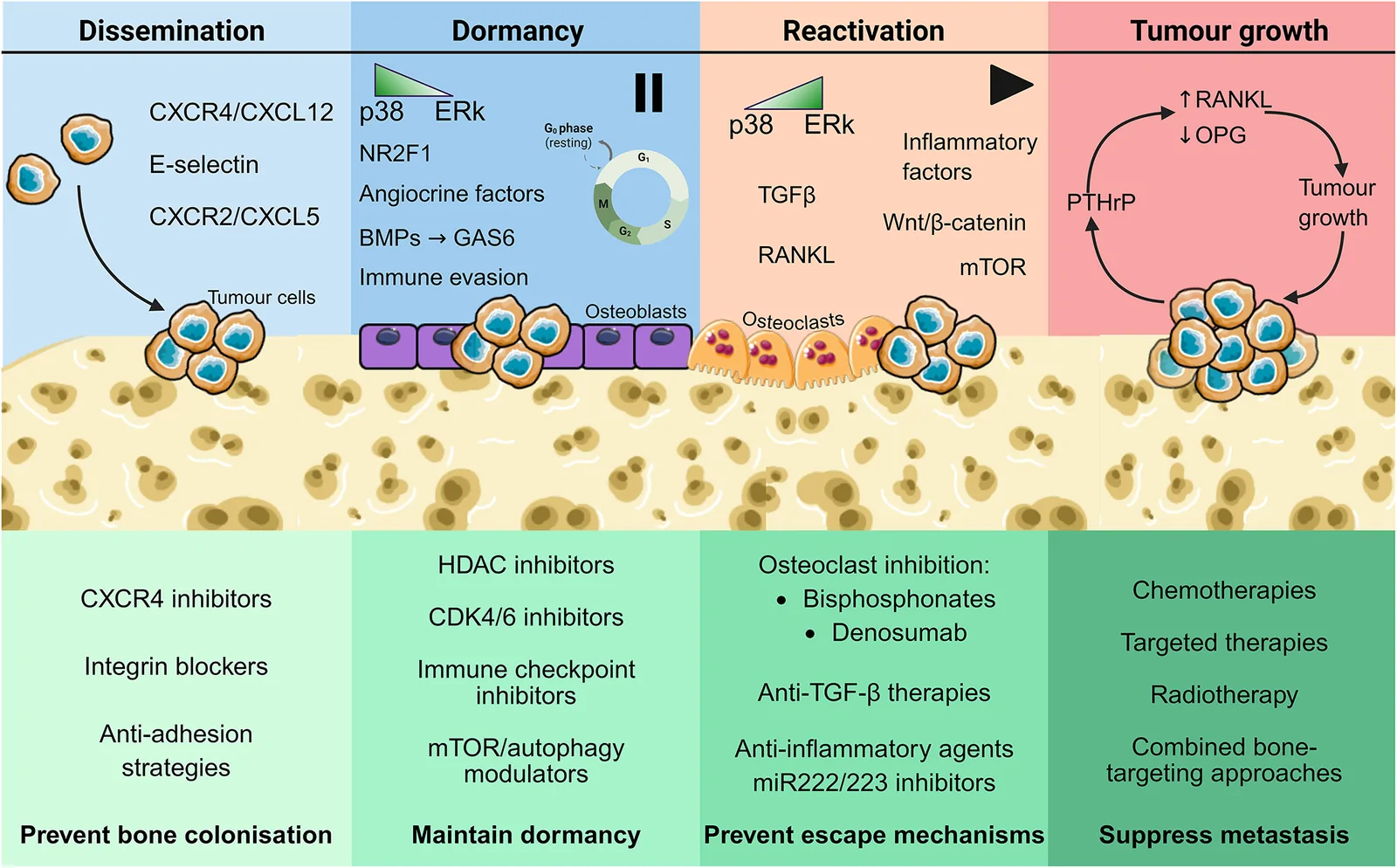
Schematic representation of breast cancer dissemination, dormancy, awakening and progression in the bone. Therapeutic strategies can target different time-points of tumour dormancy in bone, utilising the ‘sleeping’, ‘awakening’ and ‘killing’ strategies. This highlights the range of mechanisms currently under investigation, with a combination approach potentially required to target different stages of dormancy [2, 74, 83, 85, 94, 99, 111, 112, 112]. Figure created with adapted images from Servier Medical Art (http://smart.servier.com) and BioRender (https://BioRender.com/ebe4wg0), licensed under CC BY 4.0 (https://creativecommons.org/licenses/by/4.0/)

Development of effective interventions to target dormant, as well as proliferative, tumour cells is essential to prevent relapse and improve cancer survival rates. Theorietically this could be achieved by targeting any of the four distinct stages of metastatic progression: dissemination, dormancy, reactivation or tumour growth (Fig. 5). Bone colonisation of tumour cells could be prevented by utilising anti-adhesion strategies such as CXCR4 inhibitors. However, DTCs can be detected in > 60% of patients with early breast cancer [10–16], suggesting dissemination occurs extremely early on in tumour development, limiting the value of this therapeutic strategy. Efforts are being made to maintain tumour dormancy in patients, or prevent escape mechanisms, often referred to as the ‘sleeping’ strategy [196, 197], to prevent proliferative growth and metastasis using drugs such as HDAC inhibitors [85–87], or NR2F1 agonists [66–68, 198]. While this presents promise, a reservoir of disease remains in the patient, presenting a significant risk of dormant cell awakening through alternative mechanisms not being targeted. Furthermore, clonal heterogeneity and therapeutic resistance are established drivers of treatment failures, as seen with CDK4/6 inhibitors [73].

Suppressing metastasis appears to be one of the most promising approaches, using the ‘awakening’ strategy, re-awakening dormant tumour cells, sensitising them to chemotherapy-based treatments, or the ‘killing’ strategy, eliminating dormant tumour cells whilst in their quiescent state [196, 197]. However, many current methods such as chemotherapies and combined bone-targeting approaches are largely focused on reducing tumour burden once progression has begun, addressing only part of the problem. Eliminating dormant tumour cells whilst in their quiescence state would provide a huge advancement in therapeutics, yet this is still limited by difficulties in the detection of dormant cells, as effective treatment would be difficult to confirm. The use of nanotechnology delivery systems is of current interest and is being investigated for several solid tumours [199, 200]. This targeting strategy may improve current efforts to reach lesions and DTCs buried deep within the bone [201–203].

## Conclusion

The complicated interplay between cancer cells and the bone microenvironment provides a daunting barrier to long-term survival for patients with breast cancer bone metastasis. This review details a wide range of mechanisms used by tumour cells to metastasise and the impact of different pathways on tumour dormancy. It is clear from pre-clinical evidence that distinct mechanisms are used between cancer types and metastatic sites, removing the possibility of a ‘one-size fits all’ approach. Several major questions remain. What markers or landmarks can be used to identify dormant tumour cells, as opposed to slow-cycling or therapy-resistant cells? How does spatial heterogeneity and tissue cell distribution impact dormant tumour cell behaviour and the timeline of their awakening? What, if any, combination of triggers is required to promote escape from dormancy and what drives the variability between these latency periods?

Moving forward, the field must transition from identifying general pathways to determining the best approaches to target these pathways for therapeutic development. The development of biomarkers for dormant tumour cells in bone is essential in detecting dormant cells before progression, enabling early intervention. In parallel, refining a ‘sleeping strategy’ approach to therapeutics remains a highly promising path toward improving long-term outcomes for patients with breast cancer bone metastasis. Leveraging current technological advances in modelling tumour cell dormancy in bone is key to the success of further studies and therapeutic approaches.

## Data Availability

No datasets were generated or analysed during the current study.

## References

[1] Chaffer CL, Weinberg RA (2011) A perspective on cancer cell metastasis. Science 331:1559–1564 21436443 10.1126/science.1203543

[2] Neophytou CM, Kyriakou TC, Papageorgis P (2019) Mechanisms of metastatic tumor dormancy and implications for cancer therapy. Int J Mol Sci 20:6158 31817646 10.3390/ijms20246158 PMC6940943

[3] Oeztuerk-Winder F, Ventura JJ (2012) The many faces of p38 mitogen-activated protein kinase in progenitor/stem cell differentiation. Biochem J 445:1–10 22702973 10.1042/BJ20120401

[4] Pulido C et al (2017) Bone metastasis risk factors in breast cancer. eCancer 11:715 10.3332/ecancer.2017.715 PMC5295847 28194227

[5] Clements ME, Johnson RW (2019) Breast cancer dormancy in bone. Curr Osteoporos Rep 17:353–361 31468498 10.1007/s11914-019-00532-y PMC6819229

[6] Malladi S et al (2016) Metastatic latency and immune evasion through autocrine inhibition of WNT. Cell 165:45–60 27015306 10.1016/j.cell.2016.02.025 PMC4808520

[7] Roy R et al (2022) Escape from breast tumor dormancy: the convergence of obesity and menopause. Proc Natl Acad Sci U S A 119:e2204758119 36191215 10.1073/pnas.2204758119 PMC9564105

[8] Pantel K, Brakenhoff RH (2004) Dissecting the metastatic cascade. Nat Rev Cancer 4:448–456 15170447 10.1038/nrc1370

[9] Sosa MS, Avivar-Valderas A, Bragado P, Wen HC, Aguirre-Ghiso JA (2011) ERK1/2 and p38alpha/beta signaling in tumor cell quiescence: opportunities to control dormant residual disease. Clin Cancer Res 17:5850–5857 21673068 10.1158/1078-0432.CCR-10-2574 PMC3226348

[10] Klein CA (2008) The direct molecular analysis of metastatic precursor cells in breast cancer: a chance for a better understanding of metastasis and for personalised medicine. Eur J Cancer 44:2721–2725 19022661 10.1016/j.ejca.2008.09.035

[11] Hosseini H et al (2016) Early dissemination seeds metastasis in breast cancer. Nature 540:552–558 27974799 10.1038/nature20785 PMC5390864

[12] Johnson RW, Schipani E, Giaccia AJ (2015) HIF targets in bone remodeling and metastatic disease. Pharmacol Ther 150:169–177 25681658 10.1016/j.pharmthera.2015.02.002 PMC4414805

[13] Mayhew V, Omokehinde T, Johnson RW (2019) Tumor dormancy in bone. Cancer Rep 3:e1156 10.1002/cnr2.1156 PMC7337256 32632400

[14] Boxer DI, Todd CEC, Coleman R, Fogelman I (1989) Bone secondaries in breast cancer: the solitary metastasis. J Nucl Med 30:1318–1320 2754488

[15] Shirazi PH, Rayudu GVS, Fordham EW (1974) 18F bone scanning: review of indications and results of 1,500 scans. Radiology 112:257–512 4835032 10.1148/112.2.361

[16] Abrams HL (1950) Skeletal metastases in carcinoma. Radiology 55:477–640 14781359 10.1148/55.4.534

[17] Sosa MS, Bragado P, Aguirre-Ghiso JA (2014) Mechanisms of disseminated cancer cell dormancy: an awakening field. Nat Rev Cancer 14:611–622 25118602 10.1038/nrc3793 PMC4230700

[18] Quayle L, Ottewell PD, Holen I (2015) Bone metastasis: molecular mechanisms implicated in tumour cell dormancy in breast and prostate cancer. Curr Cancer Drug Targets 15:469–480 25968899 10.2174/1568009615666150506092443

[19] Haider M-T, Holen I, Dear TN, Hunter K, Brown HK (2014) Modifying the osteoblastic niche with zoledronic acid *in vivo*—potential implications for breast cancer bone metastasis. Bone 66:240–250 24971713 10.1016/j.bone.2014.06.023 PMC4127787

[20] Chatterjee S, Behnam Azad B, Nimmagadda S (2014) The intricate role of CXCR4 in cancer. Adv Cancer Res 124:31–82 25287686 10.1016/B978-0-12-411638-2.00002-1 PMC4322894

[21] Jung Y et al (2006) Regulation of SDF-1 (CXCL12) production by osteoblasts; a possible mechanism for stem cell homing. Bone 38:497–508 16337237 10.1016/j.bone.2005.10.003

[22] Shiozawa Y, Havens AM, Pienta KJ, Taichman RS (2008) The bone marrow niche: habitat to hematopoietic and mesenchymal stem cells, and unwitting host to molecular parasites. Leukemia 22:941–950 18305549 10.1038/leu.2008.48 PMC5944299

[23] Chen F, Han Y, Kang Y (2021) Bone marrow niches in the regulation of bone metastasis. Br J Cancer 124:1912–1920 33758331 10.1038/s41416-021-01329-6 PMC8184962

[24] Wang J, Loberg R, Taichman RS (2006) The pivotal role of CXCL12 (SDF-1)/CXCR4 axis in bone metastasis. Cancer Metastasis Rev 25:573–587 17165132 10.1007/s10555-006-9019-x

[25] Moharita AL et al (2006) SDF-1alpha regulation in breast cancer cells contacting bone marrow stroma is critical for normal hematopoiesis. Blood 108:3245–3252 16857992 10.1182/blood-2006-01-017459

[26] Price TT et al (2016) Dormant breast cancer micrometastases reside in specific bone marrow niches that regulate their transit to and from bone. Sci Transl Med 8:340ra73 27225183 10.1126/scitranslmed.aad4059 PMC8722465

[27] Souchak J, Mohammed NBB, Lau LS, Dimitroff CJ (2024) The role of galectins in mediating the adhesion of circulating cells to vascular endothelium. Front Immunol 15:1395714 38840921 10.3389/fimmu.2024.1395714 PMC11150550

[28] Romero-Moreno R et al (2019) The CXCL5/CXCR2 axis is sufficient to promote breast cancer colonization during bone metastasis. Nat Commun 10:4404 31562303 10.1038/s41467-019-12108-6 PMC6765048

[29] Sundfeldt K et al (1997) E-cadherin expression in human epithelial ovarian cancer and normal ovary. Int J Cancer 74:275–280 9221804 10.1002/(sici)1097-0215(19970620)74:3<275::aid-ijc7>3.0.co;2-w

[30] Wells A, Yates C, Shepard CR (2008) E-cadherin as an indicator of mesenchymal to epithelial reverting transitions during the metastatic seeding of disseminated carcinomas. Clin Exp Metastasis 25:621–628 18600305 10.1007/s10585-008-9167-1 PMC2929356

[31] Aouad P et al (2022) Epithelial-mesenchymal plasticity determines estrogen receptor positive breast cancer dormancy and epithelial reconversion drives recurrence. Nat Commun 13:4975 36008376 10.1038/s41467-022-32523-6 PMC9411634

[32] De Craene B, Berx G (2013) Regulatory networks defining EMT during cancer initiation and progression. Nat Rev Cancer 13:97–110 23344542 10.1038/nrc3447

[33] Mbalaviele G et al (1996) E-cadherin expression in human breast cancer cells suppresses the development of osteolytic bone metastases in an experimental metastasis model1. Cancer Res 56:4063–4070 8752180

[34] Assoian RK, Klein EA (2008) Growth control by intracellular tension and extracellular stiffness. Trends Cell Biol 18:347–352 18514521 10.1016/j.tcb.2008.05.002 PMC2888483

[35] Desgrosellier JS, Cheresh DA (2010) Integrins in cancer: biological implications and therapeutic opportunities. Nat Rev Cancer 10:9–22 20029421 10.1038/nrc2748 PMC4383089

[36] Meenakshi Sundaram DN, Kucharski C, Parmar MB, Kc RB, Uludağ H (2017) Polymeric delivery of siRNA against integrin-β1 (CD29) to reduce attachment and migration of breast cancer cells. Macromol Biosci 17:1600430 10.1002/mabi.201600430 28160423

[37] Li Y et al (2015) Genetic depletion and pharmacological targeting of αv integrin in breast cancer cells impairs metastasis in zebrafish and mouse xenograft models. Breast Cancer Res 17:28 25849225 10.1186/s13058-015-0537-8 PMC4381510

[38] Carter RZ et al (2015) Tumour but not stromal expression of β3 integrin is essential, and is required early, for spontaneous dissemination of bone-metastatic breast cancer. J Pathol 235:760–772 25430721 10.1002/path.4490

[39] Arnold S et al (2022) Fra-2 overexpression upregulates pro-metastatic cell-adhesion molecules, promotes pulmonary metastasis, and reduces survival in a spontaneous xenograft model of human breast cancer. J Cancer Res Clin Oncol 148:1525–1542 34693476 10.1007/s00432-021-03812-2 PMC9114065

[40] Zhao Y et al (2007) Tumor αvβ3 integrin is a therapeutic target for breast cancer bone metastases. Cancer Res 67:5821–5830 17575150 10.1158/0008-5472.CAN-06-4499

[41] Inman CK, Shore P (2003) The osteoblast transcription factor Runx2 is expressed in mammary epithelial cells and mediates osteopontin expression. J Biol Chem 278:48684–48689 14506237 10.1074/jbc.M308001200

[42] Leong DT et al (2010) Cancer-related ectopic expression of the bone-related transcription factor RUNX2 in non-osseous metastatic tumor cells is linked to cell proliferation and motility. Breast Cancer Res BCR 12:R89 21029421 10.1186/bcr2762 PMC3096982

[43] Li X-Q, Lu J-T, Tan C-C, Wang Q-S, Feng Y-M (2016) RUNX2 promotes breast cancer bone metastasis by increasing integrin α5-mediated colonization. Cancer Lett. 10.1016/j.canlet.2016.06.007 27317874 10.1016/j.canlet.2016.06.007

[44] Pantano F et al (2021) Integrin alpha5 in human breast cancer is a mediator of bone metastasis and a therapeutic target for the treatment of osteolytic lesions. Oncogene 40:1284–1299 33420367 10.1038/s41388-020-01603-6 PMC7892344

[45] Barney LE et al (2020) Tumor cell–organized fibronectin maintenance of a dormant breast cancer population. Sci Adv 6:eaaz4157 32195352 10.1126/sciadv.aaz4157 PMC7065904

[46] Kiwanuka E et al (2013) CCN2 promotes keratinocyte adhesion and migration via integrin α5β1. Exp Cell Res 319:2938–2946 23988606 10.1016/j.yexcr.2013.08.021

[47] Mierke CT, Frey B, Fellner M, Herrmann M, Fabry B (2011) Integrin α5β1 facilitates cancer cell invasion through enhanced contractile forces. J Cell Sci 124:369–383 21224397 10.1242/jcs.071985 PMC3021998

[48] Dai B, Clark AM, Wells A (2024) Mesenchymal stem cell-secreted exosomes and soluble signals regulate breast cancer metastatic dormancy: current progress and future outlook. Int J Mol Sci 25:7133 39000239 10.3390/ijms25137133 PMC11241820

[49] Ono M et al (2014) Exosomes from bone marrow mesenchymal stem cells contain a microRNA that promotes dormancy in metastatic breast cancer cells. Sci Signal 7:ra63 24985346 10.1126/scisignal.2005231

[50] Bartosh TJ, Ullah M, Zeitouni S, Beaver J, Prockop DJ (2016) Cancer cells enter dormancy after cannibalizing mesenchymal stem/stromal cells (MSCs). Proc Natl Acad Sci U S A 113:E6447–E6456 27698134 10.1073/pnas.1612290113 PMC5081643

[51] Fiori ME et al (2019) Cancer-associated fibroblasts as abettors of tumor progression at the crossroads of EMT and therapy resistance. Mol Cancer 18:70 30927908 10.1186/s12943-019-0994-2 PMC6441236

[52] Liang Y, Chen W-M, Zhang Y, Li L (2026) Remodeling the tumor dormancy ecosystem to prevent recurrence and metastasis. Signal Transduct Target Ther 11:1 41484089 10.1038/s41392-025-02328-2 PMC12764966

[53] Giger NV, Wehrle E (2026) Advances in spatial transcriptomics in bone. Curr Osteoporos Rep 24:3 41535622 10.1007/s11914-025-00949-8 PMC12804215

[54] Sawant A, Ponnazhagan S (2013) Myeloid-derived suppressor cells as osteoclast progenitors: a novel target for controlling osteolytic bone metastasis. Cancer Res 73:4606–4610 23887974 10.1158/0008-5472.CAN-13-0305 PMC3732563

[55] Ghajar CM et al (2013) The perivascular niche regulates breast tumour dormancy. Nat Cell Biol 15:807–817 23728425 10.1038/ncb2767 PMC3826912

[56] Kawano T et al (2019) Temporal and spatial profile of polymorphonuclear myeloid-derived suppressor cells (PMN-MDSCs) in ischemic stroke in mice. PLoS ONE 14:e0215482 31048856 10.1371/journal.pone.0215482 PMC6497247

[57] Schuettpelz LG, Link DC (2011) Niche competition and cancer metastasis to bone. J Clin Invest 121:1253–1255 21436576 10.1172/JCI57229 PMC3069798

[58] Bliss SA et al (2016) Mesenchymal stem cell-derived exosomes stimulate cycling quiescence and early breast cancer dormancy in bone marrow. Cancer Res 76:5832–5844 27569215 10.1158/0008-5472.CAN-16-1092

[59] Sandiford OA et al (2021) Mesenchymal stem cell-secreted extracellular vesicles instruct stepwise dedifferentiation of breast cancer cells into dormancy at the bone marrow perivascular region. Cancer Res 81:1567–1582 33500249 10.1158/0008-5472.CAN-20-2434

[60] Lim PK et al (2011) Gap junction-mediated import of MicroRNA from bone marrow stromal cells can elicit cell cycle quiescence in breast cancer cells. Cancer Res 71:1550–1560 21343399 10.1158/0008-5472.CAN-10-2372

[61] Adam AP et al (2009) Computational identification of a p38SAPK-regulated transcription factor network required for tumor cell quiescence. Cancer Res 69:5664–5672 19584293 10.1158/0008-5472.CAN-08-3820 PMC2720524

[62] Nobre AR et al (2021) Bone marrow NG2(+)/Nestin(+) mesenchymal stem cells drive DTC dormancy via TGFbeta2. Nat Cancer 2:327–339 34993493 10.1038/s43018-021-00179-8 PMC8730384

[63] Gawrzak S et al (2018) MSK1 regulates luminal cell differentiation and metastatic dormancy in ER(+) breast cancer. Nat Cell Biol 20:211–221 29358704 10.1038/s41556-017-0021-z

[64] Jönsson M, Dejmek J, Bendahl P-O, Andersson T (2002) Loss of Wnt-5a protein is associated with early relapse in invasive ductal breast Carcinomas1. Cancer Res 62:409–416 11809689

[65] Onitilo AA, Engel JM, Greenlee RT, Mukesh BN (2009) Breast cancer subtypes based on ER/PR and Her2 expression: comparison of clinicopathologic features and survival. Clin Med Res 7:4–13 19574486 10.3121/cmr.2009.825 PMC2705275

[66] Carnero A, Lleonart M (2016) The hypoxic microenvironment: a determinant of cancer stem cell evolution. BioEssays 38(Suppl 1):S65-74 27417124 10.1002/bies.201670911

[67] Fluegen G et al (2017) Phenotypic heterogeneity of disseminated tumour cells is preset by primary tumour hypoxic microenvironments. Nat Cell Biol 19:120–132 28114271 10.1038/ncb3465 PMC5342902

[68] Sui L, Wang J, Jiang WG, Song X, Ye L (2024) Molecular mechanism of bone metastasis in breast cancer. Front Oncol 14:1401113 39605887 10.3389/fonc.2024.1401113 PMC11599183

[69] Li C, Zhao R, Yang H, Ren L (2023) Construction of bone hypoxic microenvironment based on bone-on-a-chip platforms. Int J Mol Sci 24:6999 37108162 10.3390/ijms24086999 PMC10139217

[70] Cardoso F et al (2020) 5th ESO-ESMO international consensus guidelines for advanced breast cancer (ABC 5). Ann Oncol 31:1623–1649 32979513 10.1016/j.annonc.2020.09.010 PMC7510449

[71] Abdelmalak M et al (2022) The renaissance of CDK inhibitors in breast cancer therapy: an update on clinical trials and therapy resistance. Cancers 14:5388 36358806 10.3390/cancers14215388 PMC9655989

[72] Marei HE et al (2025) Targeting CDKs in cancer therapy: advances in PROTACs and molecular glues. Npj Precis Oncol 9:204 40581698 10.1038/s41698-025-00931-8 PMC12206236

[73] Glaviano A et al (2024) Mechanisms of sensitivity and resistance to CDK4/CDK6 inhibitors in hormone receptor-positive breast cancer treatment. Drug Resist Updat 76:101103 38943828 10.1016/j.drup.2024.101103

[74] Hassanzadeh A et al (2024) Cancer therapy by cyclin-dependent kinase inhibitors (CDKIs): bench to bedside. EXCLI J 23:862–882 38983782 10.17179/excli2024-7076 PMC11231458

[75] Wang Y, Liu L, Zhang X, Liang T, Bai X (2025) Cancer dormancy and metabolism: from molecular insights to translational opportunities. Cancer Lett 635:218097 41135858 10.1016/j.canlet.2025.218097

[76] Chaterjee M, van Golen KL (2011) Breast cancer stem cells survive periods of farnesyl-transferase inhibitor-induced dormancy by undergoing autophagy. Bone Marrow Res 2011:362938 22046561 10.1155/2011/362938 PMC3199942

[77] Hosokawa N et al (2009) Nutrient-dependent mTORC1 association with the ULK1-Atg13-FIP200 complex required for autophagy. Mol Biol Cell 20:1981–1991 19211835 10.1091/mbc.E08-12-1248 PMC2663915

[78] Vera-Ramirez L, Vodnala SK, Nini R, Hunter KW, Green JE (2018) Autophagy promotes the survival of dormant breast cancer cells and metastatic tumour recurrence. Nat Commun 9:1944 29789598 10.1038/s41467-018-04070-6 PMC5964069

[79] Kim DH et al (2002) mTOR interacts with raptor to form a nutrient-sensitive complex that signals to the cell growth machinery. Cell 110:163–175 12150925 10.1016/s0092-8674(02)00808-5

[80] Schreiber RD, Old LJ, Smyth MJ (2011) Cancer immunoediting: integrating immunity’s roles in cancer suppression and promotion. Science 331:1565–1570 21436444 10.1126/science.1203486

[81] Barrow AD et al (2018) Natural killer cells control tumor growth by sensing a growth factor. Cell 172:534-548.e19 29275861 10.1016/j.cell.2017.11.037 PMC6684025

[82] Bushnell GG et al (2024) Natural killer cell regulation of breast cancer stem cells mediates metastatic dormancy. Cancer Res 84:3337–3353 39106452 10.1158/0008-5472.CAN-24-0030 PMC11474167

[83] Hamza FN, Mohammad KS (2024) Immunotherapy in the battle against bone metastases: mechanisms and emerging treatments. Pharmaceuticals 17:1591 39770433 10.3390/ph17121591 PMC11679356

[84] Isla Larrain MT et al (2014) IDO is highly expressed in breast cancer and breast cancer-derived circulating microvesicles and associated to aggressive types of tumors by in silico analysis. Tumor Biol 35:6511–6519 10.1007/s13277-014-1859-3 24687552

[85] Clements ME, Holtslander L, Johnson JR, Johnson RW (2023) Select HDAC inhibitors enhance osteolysis and bone metastasis outgrowth but can be mitigated with bisphosphonate therapy. JBMR Plus 7:e10694 36936362 10.1002/jbm4.10694 PMC10020917

[86] Clements ME et al (2021) HDAC inhibitors induce LIFR expression and promote a dormancy phenotype in breast cancer. Oncogene 40:5314–5326 34247191 10.1038/s41388-021-01931-1 PMC8403155

[87] Johnson RW et al (2016) Induction of LIFR confers a dormancy phenotype in breast cancer cells disseminated to the bone marrow. Nat Cell Biol 18:1078–1089 27642788 10.1038/ncb3408 PMC5357601

[88] Edwards CM, Johnson RW (2021) Targeting histone modifications in bone and lung metastatic cancers. Curr Osteoporos Rep 19:230–246 33721181 10.1007/s11914-021-00670-2 PMC8316289

[89] Humphrey C et al (2023) A pilot study of the combination of entinostat with capecitabine in high-risk breast cancer after neoadjuvant therapy. J Clin Oncol 41:e13107–e13107

[90] Connolly RM et al (2021) E2112: randomized phase III trial of endocrine therapy plus entinostat or placebo in hormone receptor-positive advanced breast cancer: a trial of the ECOG-ACRIN cancer research group. J Clin Oncol 39:3171–3181 34357781 10.1200/JCO.21.00944 PMC8478386

[91] O’Shaughnessy J et al (2020) Results of ENCORE 602 (TRIO025), a phase II, randomized, placebo-controlled, double-blinded, multicenter study of atezolizumab with or without entinostat in patients with advanced triple-negative breast cancer (aTNBC). J Clin Oncol 38:1014–1014

[92] Searcy MB, Johnson RW (2024) Epigenetic control of the vicious cycle. J Bone Oncol 44:100524 38304486 10.1016/j.jbo.2024.100524 PMC10830514

[93] Zhao Y et al (2024) Long noncoding RNA Malat1 protects against osteoporosis and bone metastasis. Nat Commun 15:2384 38493144 10.1038/s41467-024-46602-3 PMC10944492

[94] Robinson NJ, Parker KA, Schiemann WP (2020) Epigenetic plasticity in metastatic dormancy: mechanisms and therapeutic implications. Ann Transl Med 8:903–903 32793747 10.21037/atm.2020.02.177 PMC7396775

[95] Wikman H, Vessella R, Pantel K (2008) Cancer micrometastasis and tumour dormancy. APMIS 116:754–770 18834417 10.1111/j.1600-0463.2008.01033.x

[96] Lu X et al (2011) VCAM-1 promotes osteolytic expansion of indolent bone micrometastasis of breast cancer by engaging alpha4beta1-positive osteoclast progenitors. Cancer Cell 20:701–714 22137794 10.1016/j.ccr.2011.11.002 PMC3241854

[97] Ottewell PD et al (2015) OPG-Fc inhibits ovariectomy-induced growth of disseminated breast cancer cells in bone. Int J Cancer 137:968–977 25603921 10.1002/ijc.29439

[98] Aapro M et al (2008) Guidance on the use of bisphosphonates in solid tumours: recommendations of an international expert panel. Ann Oncol 19:420–432 17906299 10.1093/annonc/mdm442

[99] Diel IJ (2010) Bisphosphonates in breast cancer patients with bone metastases. Breast Care 5:306–311 21779212 10.1159/000322043 PMC3132954

[100] Ottewell PD et al (2014) Zoledronic acid has differential antitumor activity in the pre- and postmenopausal bone microenvironment in vivo. Clin Cancer Res 20:2922–2932 24687923 10.1158/1078-0432.CCR-13-1246 PMC4040234

[101] Li Y-Y et al (2020) Bisphosphonates and risk of cancers: a systematic review and meta-analysis. Br J Cancer 123:1570–1581 32901134 10.1038/s41416-020-01043-9 PMC7652831

[102] Gnant M et al (2015) Adjuvant denosumab in breast cancer (ABCSG-18): a multicentre, randomised, double-blind, placebo-controlled trial. The Lancet 386:433–443 10.1016/S0140-6736(15)60995-3 26040499

[103] Stopeck AT et al (2010) Denosumab compared with zoledronic acid for the treatment of bone metastases in patients with advanced breast cancer: a randomized, Double-Blind Study. J Clin Oncol 28:5132–5139 21060033 10.1200/JCO.2010.29.7101

[104] Rosen LS et al (2003) Long-term efficacy and safety of zoledronic acid compared with pamidronate disodium in the treatment of skeletal complications in patients with advanced multiple myeloma or breast carcinoma. Cancer 98:1735–1744 14534891 10.1002/cncr.11701

[105] Amadori D et al (2013) Efficacy and safety of 12-weekly versus 4-weekly zoledronic acid for prolonged treatment of patients with bone metastases from breast cancer (ZOOM): a phase 3, open-label, randomised, non-inferiority trial. Lancet Oncol 14:663–670 23684411 10.1016/S1470-2045(13)70174-8

[106] Hortobagyi GN et al (2017) Continued treatment effect of zoledronic acid dosing every 12 vs 4 weeks in women with breast cancer metastatic to bone: the OPTIMIZE-2 randomized clinical trial. JAMA Oncol 3:906–912 28125763 10.1001/jamaoncol.2016.6316 PMC5824238

[107] Himelstein AL et al (2017) Effect of longer-interval vs standard dosing of zoledronic acid on skeletal events in patients with bone metastases: a randomized clinical trial. JAMA 317:48–58 28030702 10.1001/jama.2016.19425 PMC5321662

[108] Coleman R et al (2014) Adjuvant zoledronic acid in patients with early breast cancer: final efficacy analysis of the AZURE (BIG 01/04) randomised open-label phase 3 trial. Lancet Oncol 15:997–1006 25035292 10.1016/S1470-2045(14)70302-X

[109] Capietto A-H, Faccio R (2014) Immune regulation of bone metastasis. BoneKEy Rep 3:600 25512853 10.1038/bonekey.2014.95 PMC4260446

[110] Zhang Q et al (2012) Prognostic significance of tumor-associated macrophages in solid tumor: a meta-analysis of the literature. PLoS ONE 7:e50946 23284651 10.1371/journal.pone.0050946 PMC3532403

[111] Byrne NM, Summers MA, McDonald MM (2019) Tumor cell dormancy and reactivation in bone: skeletal biology and therapeutic opportunities. JBMR Plus 3:e10125 30918917 10.1002/jbm4.10125 PMC6419605

[112] Jiang K, Xu F (2025) Denosumab monotherapy versus bisphosphonate switching in breast cancer bone metastases: a real-world cohort study on skeletal events and safety. J Clin Oncol 43:e13166–e13166

[113] Öner İ et al (2025) A comparison of the efficacy and safety of Denosumab and Zoledronic Acid in patients with bone metastatic breast cancer receiving CDK4/6 inhibitor therapy. Medicina (Mex) 61:360 10.3390/medicina61020360 PMC11857531 40005476

[114] Amgen. A Randomized, Double-Blind, Placebo-Controlled, Multi-Center Phase 3 Study of Denosumab as Adjuvant Treatment for Women With Early-Stage Breast Cancer at High Risk of Recurrence (D-CARE). https://clinicaltrials.gov/study/NCT01077154 (2021)

[115] Guise TA et al (2002) Parathyroid hormone-related protein (PTHrP)-(1–139) isoform is efficiently secreted in vitro and enhances breast cancer metastasis to bone in vivo. Bone 30:670–676 11996903 10.1016/s8756-3282(02)00685-3

[116] Guise TA et al (1996) Evidence for a causal role of parathyroid hormone-related protein in the pathogenesis of human breast cancer-mediated osteolysis. J Clin Invest 98:1544–1549 8833902 10.1172/JCI118947 PMC507586

[117] Kang Y et al (2005) Breast cancer bone metastasis mediated by the Smad tumor suppressor pathway. Proc Natl Acad Sci U A 102:13909–13914 10.1073/pnas.0506517102 PMC1236573 16172383

[118] Gao H et al (2012) The BMP inhibitor Coco reactivates breast cancer cells at lung metastatic sites. Cell 150:764–779 22901808 10.1016/j.cell.2012.06.035 PMC3711709

[119] Weidenfeld K et al (2016) Dormant tumor cells expressing LOXL2 acquire a stem-like phenotype mediating their transition to proliferative growth. Oncotarget 7:71362–71377 27655685 10.18632/oncotarget.12109 PMC5342084

[120] Shaikat AH et al (2026) Investigating hypoxia-inducible factor signaling in cancer: Mechanisms, clinical implications, targeted therapeutic strategies, and resistance. Cancer Pathog Ther 4:174–191 41732210 10.1016/j.cpt.2025.07.003 PMC12925099

[121] Abd GM, Laird MC, Ku JC, Li Y (2023) Hypoxia-induced cancer cell reprogramming: a review on how cancer stem cells arise. Front Oncol 13:1227884 37614497 10.3389/fonc.2023.1227884 PMC10442830

[122] Butturini E, Carcereri de Prati A, Boriero D, Mariotto S (2019) Tumor dormancy and interplay with hypoxic tumor microenvironment. Int J Mol Sci 20:4305 31484342 10.3390/ijms20174305 PMC6747268

[123] Rofstad EK, Gaustad J-V, Egeland TAM, Mathiesen B, Galappathi K (2010) Tumors exposed to acute cyclic hypoxic stress show enhanced angiogenesis, perfusion and metastatic dissemination. Int J Cancer 127:1535–1546 20091868 10.1002/ijc.25176

[124] Liu Q, Palmgren VAC, Danen EH, Le Dévédec SE (2022) Acute vs. chronic vs. intermittent hypoxia in breast cancer: a review on its application in in vitro research. Mol Biol Rep 49:10961–10973 36057753 10.1007/s11033-022-07802-6 PMC9618509

[125] Kritikou EA et al (2003) A dual, non-redundant, role for LIF as a regulator of development and STAT3-mediated cell death in mammary gland. Development 130:3459–3468 12810593 10.1242/dev.00578

[126] Pires IM et al (2010) Effects of acute versus chronic hypoxia on DNA damage responses and genomic instability. Cancer Res 70:925–935 20103649 10.1158/0008-5472.CAN-09-2715 PMC2923514

[127] Bayer C, Shi K, Astner ST, Maftei C-A, Vaupel P (2011) Acute versus chronic hypoxia: why a simplified classification is simply not enough. Int J Radiat Oncol Biol Phys 80:965–968 21683887 10.1016/j.ijrobp.2011.02.049

[128] Rolli M, Fransvea E, Pilch J, Saven A, Felding-Habermann B (2003) Activated integrin αvβ3 cooperates with metalloproteinase MMP-9 in regulating migration of metastatic breast cancer cells. Proc Natl Acad Sci U S A 100:9482–9487 12874388 10.1073/pnas.1633689100 PMC170944

[129] Shah M et al (2012) An MMP13-selective inhibitor delays primary tumor growth and the onset of tumor-associated osteolytic lesions in experimental models of breast cancer. PLoS ONE 7:e29615 22253746 10.1371/journal.pone.0029615 PMC3256168

[130] Grawenda AM, O’Neill E (2015) Clinical utility of RASSF1A methylation in human malignancies. Br J Cancer 113:372–381 26158424 10.1038/bjc.2015.221 PMC4522630

[131] Mehrotra J et al (2004) Very high frequency of hypermethylated genes in breast cancer metastasis to the bone, brain, and lung. Clin Cancer Res 10:3104–3109 15131050 10.1158/1078-0432.ccr-03-0118

[132] Zhang K et al (2021) A single-cell atlas of chromatin accessibility in the human genome. Cell 184:5985-6001.e19 34774128 10.1016/j.cell.2021.10.024 PMC8664161

[133] Gao X, Xu Z (2008) Mechanisms of action of angiogenin. Acta Biochim Biophys Sin 40:619–624 18604453 10.1111/j.1745-7270.2008.00442.x

[134] Barcellos-Hoff MH, Lyden D, Wang TC (2013) The evolution of the cancer niche during multistage carcinogenesis. Nat Rev Cancer 13:511–518 23760023 10.1038/nrc3536

[135] Naumov GN et al (2006) A model of human tumor dormancy: an angiogenic switch from the nonangiogenic phenotype. J Natl Cancer Inst 98:316–325 16507828 10.1093/jnci/djj068

[136] Laurent J et al (2011) Proangiogenic factor PlGF programs CD11b+ myelomonocytes in breast cancer during differentiation of their hematopoietic progenitors. Cancer Res 71:3781–3791 21507936 10.1158/0008-5472.CAN-10-3684

[137] Mantovani A, Sozzani S, Locati M, Allavena P, Sica A (2002) Macrophage polarization: tumor-associated macrophages as a paradigm for polarized M2 mononuclear phagocytes. Trends Immunol 23:549–555 12401408 10.1016/s1471-4906(02)02302-5

[138] Biswas SK, Mantovani A (2010) Macrophage plasticity and interaction with lymphocyte subsets: cancer as a paradigm. Nat Immunol 11:889–896 20856220 10.1038/ni.1937

[139] Yin JJ et al (1999) TGF-beta signaling blockade inhibits PTHrP secretion by breast cancer cells and bone metastases development. J Clin Invest 103:197–206 9916131 10.1172/JCI3523 PMC407876

[140] Eyre R et al (2019) Microenvironmental IL1beta promotes breast cancer metastatic colonisation in the bone via activation of Wnt signalling. Nat Commun 10:5016 31676788 10.1038/s41467-019-12807-0 PMC6825219

[141] Bellavia D et al (2022) The binomial ‘inflammation-epigenetics’ in breast cancer progression and bone metastasis: IL-1β actions are influenced by TET inhibitor in MCF-7 cell line. Int J Mol Sci 23:15422 36499741 10.3390/ijms232315422 PMC9741332

[142] Sosnoski DM, Norgard RJ, Grove CD, Foster SJ, Mastro AM (2015) Dormancy and growth of metastatic breast cancer cells in a bone-like microenvironment. Clin Exp Metastasis 32:335–344 25749879 10.1007/s10585-015-9710-9

[143] Tulotta C et al (2021) IL-1B drives opposing responses in primary tumours and bone metastases; harnessing combination therapies to improve outcome in breast cancer. NPJ Breast Cancer 7:95 34290237 10.1038/s41523-021-00305-w PMC8295314

[144] Tulotta C et al (2019) Endogenous production of IL1B by breast cancer cells drives metastasis and colonization of the bone microenvironment. Clin Cancer Res 25:2769–2782 30670488 10.1158/1078-0432.CCR-18-2202

[145] Tulotta C, Ottewell P (2018) The role of IL-1B in breast cancer bone metastasis. Endocr Relat Cancer 25:R421–R434 29760166 10.1530/ERC-17-0309 PMC5987176

[146] Nutter F et al (2014) Different molecular profiles are associated with breast cancer cell homing compared with colonisation of bone: evidence using a novel bone-seeking cell line. Endocr Relat Cancer 21:327–341 24413608 10.1530/ERC-13-0158

[147] Holen I et al (2016) IL-1 drives breast cancer growth and bone metastasis in vivo. Oncotarget 7:75571–75584 27765923 10.18632/oncotarget.12289 PMC5342762

[148] Johnson RW et al (2018) Parathyroid Hormone-Related Protein negatively regulates tumor cell dormancy genes in a PTHR1/Cyclic AMP-independent manner. Front Endocrinol 9:241 10.3389/fendo.2018.00241 PMC5964132 29867773

[149] Lin JL, Wang MJ, Lee D, Liang CC, Lin S (2008) Hypoxia-inducible factor-1alpha regulates matrix metalloproteinase-1 activity in human bone marrow-derived mesenchymal stem cells. FEBS Lett 582:2615–2619 18588890 10.1016/j.febslet.2008.06.033

[150] Powell GJ et al (1991) Localization of parathyroid hormone-related protein in breast cancer metastases: increased incidence in bone compared with other sites1. Cancer Res 51:3059–3061 2032246

[151] Kane JF, Johnson RW (2023) Re-evaluating the role of PTHrP in breast cancer. Cancers 15:2670 37345007 10.3390/cancers15102670 PMC10216606

[152] Martin TJ, Johnson RW (2021) Multiple actions of PTHrP in breast cancer bone metastasis. Br J Pharmacol 178:1923–1935 31087800 10.1111/bph.14709 PMC8445224

[153] Sethi N, Dai X, Winter CG, Kang Y (2011) Tumor-derived Jagged1 promotes osteolytic bone metastasis of breast cancer by engaging Notch signaling in bone cells. Cancer Cell 19:192–205 21295524 10.1016/j.ccr.2010.12.022 PMC3040415

[154] Tahara RK, Brewer TM, Theriault RL, Ueno NT (2019) Bone metastasis of breast cancer. In: Ahmad A (ed) Breast cancer metastasis and drug resistance: challenges and progress. Springer, Cham, pp 105–129

[155] Zhang L et al (2022) EZH2 engages TGFβ signaling to promote breast cancer bone metastasis via integrin β1-FAK activation. Nat Commun 13:2543 35538070 10.1038/s41467-022-30105-0 PMC9091212

[156] Xu Y et al (2021) Circular RNA circIKBKB promotes breast cancer bone metastasis through sustaining NF-κB/bone remodeling factors signaling. Mol Cancer 20:98 34325714 10.1186/s12943-021-01394-8 PMC8320207

[157] Bakhshandeh S et al (2024) Dormancy-inducing 3D engineered matrix uncovers mechanosensitive and drug-protective FHL2-p21 signaling axis. Sci Adv 10:eadr3997 39504377 10.1126/sciadv.adr3997 PMC11540038

[158] Barkan D et al (2010) Metastatic growth from dormant cells induced by a col-I-enriched fibrotic environment. Cancer Res 70:5706–5716 20570886 10.1158/0008-5472.CAN-09-2356 PMC3436125

[159] Barkan D et al (2008) Inhibition of metastatic outgrowth from single dormant tumor cells by targeting the cytoskeleton. Cancer Res 68:6241–6250 18676848 10.1158/0008-5472.CAN-07-6849 PMC2561279

[160] Marlow R et al (2013) A novel model of dormancy for bone metastatic breast cancer cells. Cancer Res 73:6886–6899 24145351 10.1158/0008-5472.CAN-13-0991

[161] Pradhan L et al (2023) Dynamic bioinspired coculture model for probing ER+ breast cancer dormancy in the bone marrow niche. Sci Adv 9:eade3186 36888709 10.1126/sciadv.ade3186 PMC9995072

[162] Ganesh K (2020) Plasticity in motion: Shape-shifting Lgr5− cells initiate colorectal cancer metastasis. Cell Stem Cell 26:469–471 32243803 10.1016/j.stem.2020.03.007 PMC7295070

[163] Vlachogiannis G et al (2018) Patient-derived organoids model treatment response of metastatic gastrointestinal cancers. Science 359:920–926 29472484 10.1126/science.aao2774 PMC6112415

[164] Aguirre Ghiso JA, Kovalski K, Ossowski L (1999) Tumor dormancy induced by downregulation of urokinase receptor in human carcinoma involves integrin and MAPK signaling. J Cell Biol 147:89–104 10508858 10.1083/jcb.147.1.89 PMC2164973

[165] Aguirre-Ghiso JA, Estrada Y, Liu D, Ossowski L (2003) ERK(MAPK) activity as a determinant of tumor growth and dormancy; regulation by p38(SAPK). Cancer Res 63:1684–1695 12670923

[166] Aguirre-Ghiso JA, Liu D, Mignatti A, Kovalski K, Ossowski L (2001) Urokinase receptor and fibronectin regulate the ERK(MAPK) to p38(MAPK) activity ratios that determine carcinoma cell proliferation or dormancy in vivo. Mol Biol Cell 12:863–879 11294892 10.1091/mbc.12.4.863 PMC32272

[167] Han W et al (2021) *In vitro* bone metastasis dwelling in a 3D bioengineered niche. Biomaterials 269:120624 33421710 10.1016/j.biomaterials.2020.120624

[168] Rao SS, Kondapaneni RV, Narkhede AA (2019) Bioengineered models to study tumor dormancy. J Biol Eng 13:3 30647771 10.1186/s13036-018-0137-0 PMC6327399

[169] González Díaz EC, Tai M, Monette CEF, Wu JY, Yang F (2023) Spatially patterned 3D model mimics key features of cancer metastasis to bone. Biomaterials 299:122163 37236137 10.1016/j.biomaterials.2023.122163 PMC10621670

[170] Touny LHE et al (2014) Combined SFK/MEK inhibition prevents metastatic outgrowth of dormant tumor cells. J Clin Invest 124:156–168 24316974 10.1172/JCI70259 PMC3871237

[171] Hao S et al (2018) A spontaneous 3D bone-on-a-chip for bone metastasis study of breast cancer cells. Small 14:1702787 10.1002/smll.201702787 29399951

[172] Bersini S et al (2014) A microfluidic 3D *in vitro* model for specificity of breast cancer metastasis to bone. Biomaterials 35:2454–2461 24388382 10.1016/j.biomaterials.2013.11.050 PMC3905838

[173] Jeon JS et al (2015) Human 3D vascularized organotypic microfluidic assays to study breast cancer cell extravasation. Proc Natl Acad Sci 112:214–219 25524628 10.1073/pnas.1417115112 PMC4291627

[174] Clark AM et al (2018) A model of dormant-emergent metastatic breast cancer progression enabling exploration of biomarker signatures*. Mol Cell Proteomics 17:619–630 29353230 10.1074/mcp.RA117.000370 PMC5880110

[175] Sharifi F et al (2020) A hepatocellular carcinoma–bone metastasis-on-a-chip model for studying thymoquinone-loaded anticancer nanoparticles. Bio-Des Manuf 3:189–202

[176] Khoon CS (2015) Experimental models of bone metastasis: Opportunities for the study of cancer dormancy. Adv Drug Deliv Rev 94:141–150 25572003 10.1016/j.addr.2014.12.007

[177] Torisawa Y et al (2014) Bone marrow–on–a–chip replicates hematopoietic niche physiology in vitro. Nat Methods 11:663–669 24793454 10.1038/nmeth.2938

[178] Marino S, Staines KA, Brown G, Howard-Jones RA, Adamczyk M (2016) Models of ex vivo explant cultures: applications in bone research. BoneKEy Rep 5:818 27408711 10.1038/bonekey.2016.49 PMC4926536

[179] Laranga R et al (2020) Trends in bone metastasis modeling. Cancers 12:2315 32824479 10.3390/cancers12082315 PMC7464021

[180] Bellido T, Delgado Calle J (2020) Ex vivo organ cultures as models to study bone biology. JBMR Plus 4:e10345 10.1002/jbm4.10345 PMC7059827 32161838

[181] Cramer EEA, Ito K, Hofmann S (2021) Ex vivo bone models and their potential in preclinical evaluation. Curr Osteoporos Rep 19:75–87 33428030 10.1007/s11914-020-00649-5 PMC7935733

[182] Sowder ME, Johnson RW (2018) Enrichment and detection of bone disseminated tumor cells in models of low tumor burden. Sci Rep 8:14299 30250146 10.1038/s41598-018-32653-2 PMC6155169

[183] Turrell FK et al (2023) Age-associated microenvironmental changes highlight the role of PDGF-C in ER+ breast cancer metastatic relapse. Nat Cancer 4:468–484 36914817 10.1038/s43018-023-00525-y PMC10132974

[184] Ma D et al (2021) Patient-derived xenograft culture-transplant system for investigation of human breast cancer metastasis. Commun Biol 4:1268 34741115 10.1038/s42003-021-02596-y PMC8571269

[185] Entenberg D, Oktay MH, Condeelis JS (2023) Intravital imaging to study cancer progression and metastasis. Nat Rev Cancer 23:25–42 36385560 10.1038/s41568-022-00527-5 PMC9912378

[186] Entenberg D et al (2011) Setup and use of a two-laser multiphoton microscope for multichannel intravital fluorescence imaging. Nat Protoc 6:1500–1520 21959234 10.1038/nprot.2011.376 PMC4028841

[187] Sipkins DA et al (2005) In vivo imaging of specialized bone marrow endothelial microdomains for tumour engraftment. Nature 435:969–973 15959517 10.1038/nature03703 PMC2570168

[188] Lee SH et al (2018) Real-time monitoring of cancer cells in live mouse bone marrow. Front Immunol 9:1681 30116236 10.3389/fimmu.2018.01681 PMC6082970

[189] Hansen-Algenstaedt N et al (2005) Femur window—a new approach to microcirculation of living bone in situ. J Orthop Res 23:1073–1082 15890486 10.1016/j.orthres.2005.02.013

[190] Jinnah AH, Zacks BC, Gwam CU, Kerr BA (2018) Emerging and established models of bone metastasis. Cancers 10:176 29865211 10.3390/cancers10060176 PMC6024970

[191] Zhou X, Liu J (2014) A computational model to predict bone metastasis in breast cancer by integrating the dysregulated pathways. BMC Cancer 14:618 25163697 10.1186/1471-2407-14-618 PMC4161863

[192] Newton PK et al (2015) Spatiotemporal progression of metastatic breast cancer: a Markov chain model highlighting the role of early metastatic sites. npj Breast Cancer 1:15018 28721371 10.1038/npjbcancer.2015.18 PMC5515198

[193] Li X et al. (2025) Streamlining large-scale genomic data management: Insights from the UK Biobank whole-genome sequencing data. Cell Genomics 5 10.1016/j.xgen.2025.101009 PMC12802598 40972583

[194] Cañellas-Socias A et al (2022) Metastatic recurrence in colorectal cancer arises from residual EMP1+ cells. Nature 611:603–613 36352230 10.1038/s41586-022-05402-9 PMC7616986

[195] Correia AL et al (2021) Hepatic stellate cells suppress NK cell-sustained breast cancer dormancy. Nature 594:566–571 34079127 10.1038/s41586-021-03614-z

[196] Recasens A, Munoz L (2019) Targeting cancer cell dormancy. Trends Pharmacol Sci 40:128–141 30612715 10.1016/j.tips.2018.12.004

[197] Yang S et al (2025) Towards understanding cancer dormancy over strategic hitching up mechanisms to technologies. Mol Cancer 24:47 39953555 10.1186/s12943-025-02250-9 PMC11829473

[198] Khalil BD et al (2021) An NR2F1-specific agonist suppresses metastasis by inducing cancer cell dormancy. J Exp Med 219:e20210836 34812843 10.1084/jem.20210836 PMC8614154

[199] Nguyen LNM et al (2024) The mechanisms of nanoparticle delivery to solid tumours. Nat Rev Bioeng 2:201–213 10.1038/s44222-024-00203-3 PMC7616668 39376248

[200] Moradi Kashkooli F, Soltani M, Momeni MM, Rahmim A (2021) Enhanced drug delivery to solid tumors via drug-loaded nanocarriers: an image-based computational framework. Front Oncol 11:655781 34249692 10.3389/fonc.2021.655781 PMC8264267

[201] Sun Z, Li B, Chen Z, Zhang Z (2026) Dormancy and recurrence in breast cancer bone metastasis: from mechanisms to clinical translation. Adv Biol 10:e00485 10.1002/adbi.202500485 41736249

[202] Lian X et al (2024) Bone-marrow-homing lipid nanoparticles for genome editing in diseased and malignant haematopoietic stem cells. Nat Nanotechnol 19:1409–1417 38783058 10.1038/s41565-024-01680-8 PMC11757007

[203] Liu C et al (2022) The osteogenic niche-targeted arsenic nanoparticles prevent colonization of disseminated breast tumor cells in the bone. Acta Pharm Sin B 12:364–377 35127392 10.1016/j.apsb.2021.06.012 PMC8799883

[204] Kobayashi A et al (2011) Bone morphogenetic protein 7 in dormancy and metastasis of prostate cancer stem-like cells in bone. J Exp Med 208:2641–2655 22124112 10.1084/jem.20110840 PMC3244043

[205] Shiozawa Y et al (2010) GAS6/AXL axis regulates prostate cancer invasion, proliferation, and survival in the bone marrow niche. Neoplasia 12:116–127 20126470 10.1593/neo.91384 PMC2814350

